# Deficiency of the dual ubiquitin/SUMO ligase Topors results in genetic instability and an increased rate of malignancy in mice

**DOI:** 10.1186/1471-2199-11-31

**Published:** 2010-04-29

**Authors:** Henderson Marshall, Mantu Bhaumik, Hana Aviv, Dirk Moore, Ming Yao, Jayeeta Dutta, Hussein Rahim, Murugesan Gounder, Shridar Ganesan, Ahamed Saleem, Eric Rubin

**Affiliations:** 1 Department of Pharmacology, Cancer Institute of New Jersey, Robert Wood Johnson Medical School, University of Medicine and Dentistry of New Jersey, 195 Little Albany Street, New Brunswick, NJ 08901, USA; 2 Pediatrics, Cancer Institute of New Jersey, Robert Wood Johnson Medical School, University of Medicine and Dentistry of New Jersey, 195 Little Albany Street, New Brunswick, NJ 08901, USA; 3 Pathology and Laboratory Medicine, Cancer Institute of New Jersey, Robert Wood Johnson Medical School, University of Medicine and Dentistry of New Jersey, 195 Little Albany Street, New Brunswick, NJ 08901, USA; 4 Biostatistics, Cancer Institute of New Jersey, Robert Wood Johnson Medical School, University of Medicine and Dentistry of New Jersey, 195 Little Albany Street, New Brunswick, NJ 08901, USA; 5 Merck Research Laboratories, North Wales, PA, 19454, USA

## Abstract

**Background:**

Topors is a nuclear protein that co-localizes with promyelocytic leukemia bodies and has both ubiquitin and SUMO E3 ligase activity. Expression studies implicated Topors as a tumor suppressor in various malignancies. To gain insight into the function of Topors, we generated a Topors-deficient mouse strain.

**Results:**

Mice homozygous for a mutant Topors allele exhibited a high rate of perinatal mortality and decreased lifespan. In addition, heterozygotes were found to have an increased incidence of malignancy, involving a variety of tissues. Consistent with this finding, primary embryonic fibroblasts lacking Topors exhibited an increased rate of malignant transformation, associated with aneuploidy and defective chromosomal segregation. While loss of Topors did not alter sensitivity to DNA-damaging or microtubule-targeting agents, cells lacking Topors exhibited altered pericentric heterochromatin, manifested by mislocalization of HP1α and an increase in transcription from pericentric major satellite DNA. Topors-deficient cells exhibited a transcriptional profile similar to that of cells treated with histone deacetylase inhibitors, and were resistant to the anti-proliferative effects of the histone deacetylase inhibitor trichostatin A.

**Conclusion:**

These results indicate a unique role for Topors in the maintenance of genomic stability and pericentric heterochromatin, as well as in cellular sensitivity to histone deacetylase inhibitors.

## Background

Topors is nuclear protein that is widely expressed in human tissues [1], and is the first example of protein that is capable of functioning as both a ubiquitin and SUMO E3 ligase [2-4]. Furthermore, expression analyses and genetic studies have implicated TOPORS as a tumor suppressor in colon, lung, brain, and prostate malignancies [5,1,6].

In proliferating cells, Topors localizes in nuclear foci, many of which co-localize with PML nuclear bodies [7]. While identified originally as a topoisomerase I- and p53-binding protein, human Topors and a *Drosophila *ortholog were shown to function as RING-dependent E3 ubiquitin ligases, with substrates including p53, the Hairy transcription factor, and the homeodomain protein NKX3.1 [2,8,9]. Additional studies indicated that Topors could act as an E3 SUMO ligase for p53 and topoisomerase I, with the RING domain dispensable for this function [3,4]. Furthermore, a recent proteomic study identified several proteins involved in chromatin regulation, including Sin3A, as potential sumoylation substrates for Topors [10]. While a *Drosophila *ortholog was shown to be required for proper functioning of a chromatin insulator [11], physiologically relevant ubiquitination/sumoylation substrates and the biological role of Topors remain poorly understood.

To gain insight into the function of Topors, we generated a Topors-deficient mouse strain using a gene-trapped allele. Although mice homozygous for the mutant allele frequently died during the perinatal period, heterozygous mice appeared normal but had an increased rate of malignancy.

## Results

### Targeted disruption of *Topors *in mice

**Figure 1:**
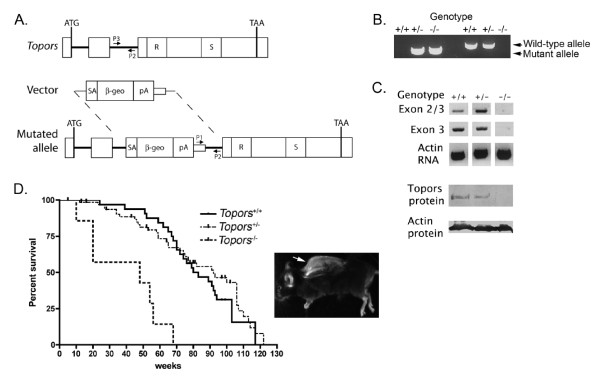
**Insertional mutation of murine *Topors***. A. Schematic of the gene-trap insertion into intron 2 of *Topors*. The three *Topors *exons are shown as rectangles. In exon 3 *R *represents the conserved RING domain required for ubiquitination activity, and *S *represents the region involved in sumoylation activity. The *SA *(splice acceptor), *β-geo *(*β-galactosidase-neomycin*) fusion gene, and *pA *(polyadenylation) sequences present in the gene-trap vector are shown. Locations of PCR primers used for genotyping are indicated by arrows. B. Representative PCR-based genotyping of mice obtained from breeding heterozygotes. C. Analysis of Topors mRNA and protein expression in colon tissue obtained from *Topors*^+/+^, *Topors*^+/-^, and *Topors*^-/- ^mice. As indicated in the Methods, RNA expression was evaluated by RT-PCR using primers spanning exons 2-3 and primers within exon 3 only. Actin RNA expression was determined using β-actin-specific primers. Protein expression was evaluated by immunoblotting nuclear lysates with a polyclonal antibody generated against the human protein, as well as a β-actin antibody. D. Kaplan-Meier survival analysis of *Topors*^+/+ ^(n = 40), *Topors*^+/- ^(n = 69), and *Topors*^-/- ^(n = 7) mice that survived weaning. The insert shows spinal kyphosis in a *Topors*^-/- ^mouse that was sacrificed at 68 weeks of age.

Topors-deficient mice were created from a gene-trapped embryonic stem cell line. The mutant cell line expresses a fusion transcript including exons 1 and 2 of *Topors*, with exon 3 replaced by vector-derived β-galactosidase sequence (Figure 1). Since exon 3 contains most of the *Topors *coding sequence (residues 69-1033), including the highly-conserved RING domain (residues 103-141) required for ubiquitination activity [2], and the 437-555 region involved in sumoylation activity [3,4], the protein derived from the fusion transcript is expected to lack both the ubiquitin and SUMO ligase activities attributed to Topors.

Mice heterozygous for the mutant allele appeared phenotypically normal and were interbred to obtain homozygotes. Analyses of Topors mRNA expression as a function of genotype indicated that similar to human tissues [1], Topors transcription was detectable in several tissues from *Topors*^+/+ ^mice, including brain, colon, kidney, and liver (data not shown). Transcripts containing exon 3 were undetectable in colon tissue obtained from mice homozygous for the mutant allele, whereas these transcripts were readily detected in colon tissue from *Topors*^+/+ ^and *Topors*^+/- ^mice (Figure 1C). Similarly, immunoblotting with a polyclonal antibody developed against the human protein [7] confirmed loss of Topors protein expression in homozygote colon tissues (Figure 1C).

Genotyping of multiple heterozygote intercrosses indicated that *Topors*^-/- ^mice were present from day E13.5 to birth at the expected Mendelian ratio (Table 1). However, *Topors*^-/- ^mice frequently died during the perinatal period and those that survived weaning were smaller than their littermates. Mean adult male weights were 60 ± 7.3 g (n = 6), 46 ± 5.8 g (n = 6), and 32.5 ± 6.5 g (n = 4) for *Topors*^+/+^, *Topors*^+/-^, and *Topors*^-/- ^mice, respectively (p = 0.0003, *Topors*^-/- ^mice compared to *Topors*^+/+ ^mice, and p = 0.009, *Topors*^-/- ^mice compared to *Topors*^+/- ^mice).

**Table 1:** Genotype Distribution of Mice Generated from Heterozygote Matings.

	Genotype			P value
	
Time of genotyping	+/+	+/-	-/-	
Day E13.5 - birth	23	58	19	0.237^a^

Weaning	16	37	7	0.051^a^

^a^Chi-square test for a non-Mendelian distribution

In addition, *Topors*^-/- ^mice that survived weaning exhibited a high rate of mortality. Predicted median survival for *Topors*^-/- ^mice that survived weaning was 48 weeks compared to 82 weeks for wild-type littermates (p < 0.0001, logrank test, Figure 1D). Necropsies of *Topors*^-/- ^mice did not disclose gross organ abnormalities or other obvious causes of death. However, spinal kyphosis was noted in two of seven *Topors*^-/- ^mice that survived weaning (these mice died at 56 and 68 weeks of age, respectively, Figure 1E). This abnormality was not detected among wild-type littermates. Both male and female *Topors*^-/- ^mice were fertile, indicating that *Topors *is not required for production of functional ova and sperm.

### Increased incidence of tumors in mice containing a mutant *Topors *allele

**Figure 2:**
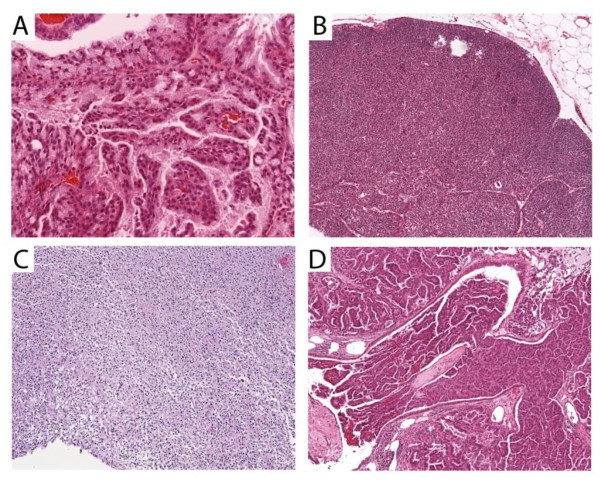
**Representative tumor histologies in *Topors*^+/- ^mice**. Representative sections of H&E-stained tissues are shown. A. Papillary Harderian gland tumor (×20). B. Thymic lymphoma (×10). C. Histiocytic sarcoma involving liver and bladder (x10). D. Bronchoalveolar adenocarcinoma of the lung (×10).

Cohorts of *Topors*^+/+^, *Topors*^+/-^, and *Topors*^-/- ^mice were monitored for several months to investigate the incidence of tumor formation. In addition, necropsies were performed on all mice in these cohorts that died, to determine whether malignancy was present. A single tumor (papillary adenoma of the Harderian gland) was detected among 40 *Topors*^+/+ ^mice (2.5% incidence). By contrast, among 69 *Topors*^+/- ^mice, 12 tumors (17% incidence) of various histologies were identified in typically older mice, and occurred in both males and females (Table 2, Figure 2). Compared to *Topors*^+/+ ^mice, *Topors*^+/- ^mice had a 7-fold increase in risk of tumor development (95% confidence interval 0.94-51.5, p = 0.0167, Fisher's Exact Test). This finding indicates that haploinsufficiency of *Topors *is associated with an increased rate of malignancy in mice.

**Table 2:** Tumors in *Topors*^+/- ^mice.

Number	Age (weeks)	Sex	Histology
1434	46	M	thymic lymphoma
1445	59	M	liver hemangioma
1519	62	M	poorly differentiated malignancy of kidney and spleen
1506	64	F	splenic lymphoma
1419	75	F	papillary Harderian gland tumor
1438	77	F	cervical lymph node lymphoma
1421	91	F	Histiocytic sarcoma of liver and bladder
1467	92	M	lung carcinoma, prostate adenocarcinoma
1426	93	F	abdominal lymphocytic lymphoma
1420	106	M	bronchioalveolar adenocarcinoma
1418	110	M	Histiocytic sarcoma of bladder
1401	117	M	bronchial adenoma

A small hepatoma was found in a *Topors*^-/- ^mouse that died at 48 weeks of age. No malignancies were detected during necropsy of three other *Topors*^-/- ^mice that survived beyond 24 weeks (ages at death were 54, 56, and 68 weeks of age, respectively). Since the median age at which tumors were detected in *Topors*^+/- ^mice was 77 weeks, the high perinatal mortality rate in *Topors*^-/- ^mice may have confounded manifestation of an increase in cancer incidence in adult *Topors*^-/- ^mice.

### Topors-deficient primary embryonic fibroblasts exhibit slow growth and genetic instability

**Figure 3:**
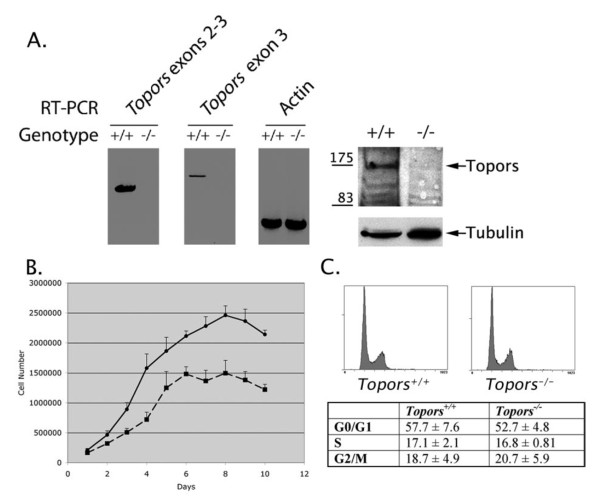
**Analysis of growth rates and cell cycle distribution in *Topors*^+/+ ^and *Topors*^-/- ^pMEFs**. **A**. Expression of Topors RNA and protein in early passage pMEFs. Lysates were obtained from pMEF cell lines of the indicated genotype. As indicated, RNA expression was evaluated by RT-PCR, using primers spanning *Topors *exons 2-3, or within exon 3 alone. Actin RNA expression was evaluated using β-actin-specific primers. The right panel shows Topors protein expression evaluated by immunoblotting nuclear lysates with a polyclonal antibody generated against the human protein. Tubulin immunoblotting is shown as a control for relative protein content. **B**. Cellular proliferation was assayed in triplicate in *Topors*^+/+ ^(solid line with circles) and *Topors*^-/- ^(dotted line with squares) early passage pMEFs. **C**. Representative cell cycle distributions as determined using propidium iodide staining and flow cytometry. The table lists mean and standard deviations of cell cycle distribution data obtained from two independent pMEF cell lines. Statistical analyses indicated no significant differences in mean values of cell cycle phases.

To investigate the cellular role of *Topors*, as well as mechanisms underlying the increased rate of malignancy observed in mice with a mutant *Topors *allele, we analyzed early passage, Topors-deficient E13.5 primary murine embryonic fibroblasts (pMEFs). Expression of Topors RNA and protein was detectable in *Topors*^+/+ ^pMEFs, but not in *Topors*^-/- ^pMEFs (Figure 3A). Early passage *Topors*^-/- ^pMEFs exhibited a slower growth rate and reached plateau earlier than wild-type cells (Figure 3B). Calculated doubling time for the *Topors*^+/+ ^cells was 1.1 days versus 1.6 days for *Topors*^-/- ^cells (p = 0.03, F test). Cell cycle analyses indicated that deficiency of Topors had little effect on cell cycle distribution (Figure 3C).

**Figure 4:**
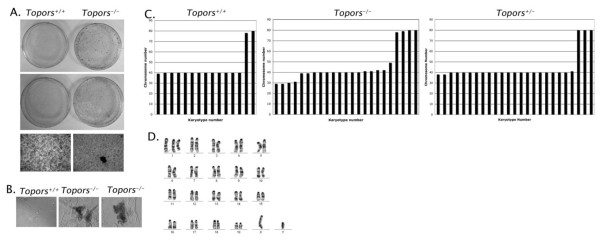
**Cellular transformation and chromosomal instability in Topors-deficient primary murine embryonic fibroblasts**. A. Formation of foci by *Topors*^-/- ^pMEFs after 3 weeks of growth. The upper section shows two representative plates for *Topors*^+/+ ^and *Topors*^-/- ^pMEFs. The lower section shows 10X-magnified images, including a focus in *Topors*^-/- ^pMEFs. B. Anchorage-independent growth by *Topors*^-/- ^pMEFs. 5 × 10^4 ^cells were seeded in soft agar and colonies assessed after 2 weeks of growth. Shown are two of four colonies detected in *Topors*^-/- ^pMEF cultures. No colonies were observed in wild-type cultures. C. Distribution of chromosome numbers for metaphases analyzed in two independent *Topors*^+/+ ^(n = 15), *Topors*^-/- ^(n = 23), and *Topors*^+/- ^(n = 25) early passage pMEF cell lines. Each bar represents a single metaphase. The difference between aneuploidy frequency in *Topors*^+/+ ^versus *Topors*^-/- ^pMEFs is statistically significant (p = 0.016). D. Representative karyotype of an early passage *Topors*^-/- ^pMEF cell, showing trisomy for chromosome 1.

To investigate whether loss of Topors was associated with an increase in the rate of cellular transformation, we examined the ability of *Topors*^-/- ^pMEFs to form foci on a monolayer of cells, and to form colonies in soft agar. In contrast to wild-type cells, *Topors*^-/- ^pMEFs frequently obtained an ability to grow as foci (Figure 4). Similarly, *Topors*^-/- ^pMEFs were capable of anchorage-independent growth in soft agar (Figure 4). These results are consistent with Topors functioning as a tumor suppressor.

Since loss of Topors results in a decrease in cellular proliferation in pMEFs (Figure 3), it is unlikely that Topors functions as a "gatekeeper"-type tumor suppressor [12]. To investigate whether loss of Topors affected genetic stability (i.e. whether Topors functions as a "caretaker"-type tumor suppressor), we analyzed karyotypes in early passage pMEFs. Among 15 *Topors*^+/+ ^cell metaphases, 13 (87%) contained the expected diploid number of 40 chromosomes, with one exhibiting tetraploidy, and only two exhibiting aneuploidy (13% aneuploidy rate, Figure 4). By contrast, 13 of 23 (56%) metaphases from *Topors*^-/- ^cells were neither diploid nor tetraploid, with chromosome counts ranging from 29 to 79 (Figure 4, p = 0.016 compared to *Topors*^+/+ ^cells aneuploidy rate, Fisher's Exact Test). When metaphases from *Topors*^+/- ^cells were examined, the aneuploidy rate was found to be similar to that of *Topors*^+/+ ^cells, with 3/25 (12%) of metaphases exhibiting aneuploidy (Figure 4).

**Figure 5:**
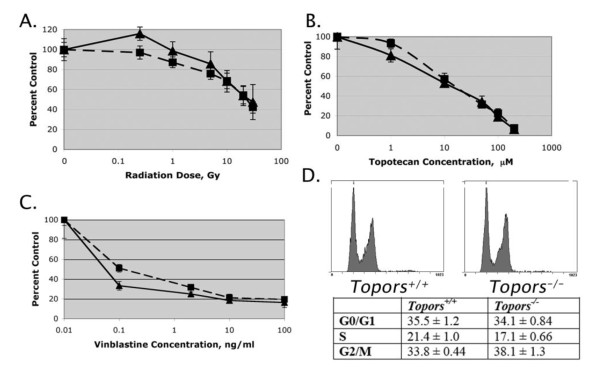
**Assessment of the sensitivity of *Topors*^+/+ ^and *Topors*^-/- ^pMEFs to DNA-damaging agents and the microtubule-targeting drug vinblastine**. **A-C**. Early passage *Topors*^+/+ ^(solid lines, triangles) and *Topors*^-/- ^(dotted lines, squares) pMEFs were exposed to increasing doses of ionizing radiation or incubated for 72 hours with increasing concentrations of topotecan or vinblastine. Anti-proliferative activity was determined using an MTT assay. Data represent means and standard deviations of 3-6 replicates. **D**. Representative cell cycle distributions observed 12 hours after vinblastine exposure, using propidium iodide staining and flow cytometry. The lower panel indicates mean and standard deviations of cell cycle distribution data obtained from two independent cell lines. Statistical analyses indicated no significant differences in mean values of cell cycle phases.

Since aneuploidy conferred by caretaker-type tumor suppressors is often related to defects in DNA repair, we analyzed the sensitivity of Topors-deficient pMEFs to ionizing radiation and to the DNA-damaging (topoisomerase I-targeting) drug topotecan. The results indicated that loss of Topors did not sensitize pMEFs to DNA damage induced by either ionizing radiation or topotecan (Figure 5). Similarly, deficiency of Topors did not result in an increase in DNA double strand breaks in untreated pMEFs as assessed by analysis of phosphorylated H2AX (γ H2AX) foci (data not shown). Furthermore, no intra-chromosomal rearrangements were detected in metaphase chromosomes from *Topors*^-/- ^cells that were analyzed using spectral karyotyping (Additional File 1: Figure S1). Together, these results indicate that deficiency of Topors results in genetic instability manifested by an increased rate of aneuploidy without an increase in sensitivity to DNA-damaging agents. These results suggest an underlying defect in chromosomal segregation, but not DNA repair, in *Topors*^-/- ^pMEFs.

Next, we investigated dysfunction of the mitotic spindle and the spindle checkpoint as possible causes of aneuploidy in Topors-deficient cells. *Topors*^+/+ ^and *Topors*^-/- ^pMEFs exhibited similar arrest in the G2/M phase of the cell cycle after a 12 hour exposure to the microtubule-targeting drug vinblastine (Figure 5). Similar were results were obtained after a 48 hour exposure to vinblastine (data not shown), and deficiency of Topors did not affect cellular sensitivity to this drug (Figure 5). These results indicate that the mitotic spindle checkpoint is intact in Topors-deficient cells. In addition, as assessed by α-tubulin immunoflourescence studies, there was no evidence of multipolar spindles or abnormal microtubule architecture in *Topors*^-/- ^pMEFs (data not shown).

### Alterations in pericentric heterochromatin and high molecular weight SUMO-2/3 conjugates in Topors-deficient primary embryonic fibroblasts

**Figure 6:**
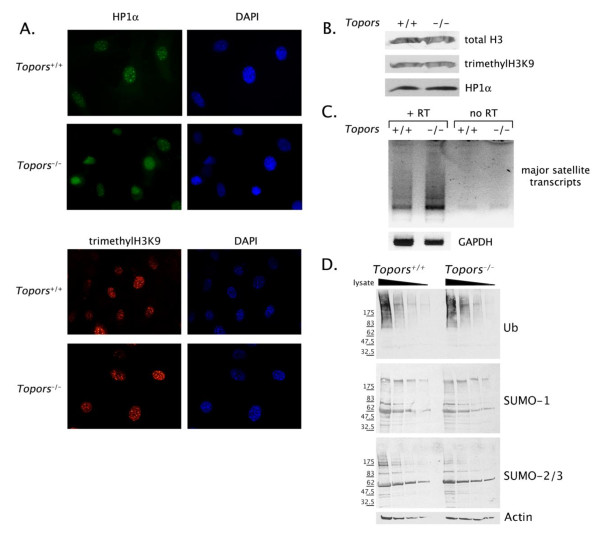
**Alterations in pericentric heterochromatin and high molecular weight SUMO-2/3 conjugates in Topors-deficient pMEFs**. **A**. Shown are representative immunofluorescence images of *Topors*^+/+ ^and *Topors*^-/- ^pMEFs using HP1α and trimethyl-H3K9 antibodies. B. Nuclear lysates from *Topors*^+/+ ^and *Topors*^-/- ^pMEFs were analyzed by immunoblotting with antibodies recognizing histone H3, trimethyl-H3K9, and HP1α as indicated. C. Analysis of pericentric major satellite repeat transcription in *Topors*^+/+ ^and *Topors*^-/- ^pMEFs. Purified RNA from *Topors*^+/+ ^and *Topors*^-/- ^pMEFs was analyzed using semi-quantitative RT-PCR and primers specific for pericentric major satellite repeat transcripts or GAPDH. For major satellite transcripts, results are shown for duplicate assays performed in the presence and absence of reverse transcriptase. The GAPDH assays were performed using a 10-fold dilution of RNA. D. Analysis of ubiquitin, SUMO-1, and SUMO-2/3 conjugates in *Topors*^+/+ ^and *Topors*^-/- ^pMEFs. Whole cell lysates were analyzed by immunoblotting with the indicated antibodies. Lanes from left to right for *Topors*^+/+ ^and *Topors*^-/- ^pMEFs indicate serial 2-fold dilutions of cellular lysates.

Alterations in pericentric chromatin are implicated in aneuploidy [13], and studies of both human and *Drosophila Topors *orthologs implicate Topors in chromatin regulation. To investigate centromeric function and pericentric heterochromatin in Topors-deficient pMEFs, we analyzed the localization of heterochromatin protein 1α (HP1α), which is associated with centromeric regions during interphase and is required for proper chromosomal segregation [14]. As expected in interphase *Topors*^+/+ ^pMEFs, HP1α was predominantly localized in DAPI-rich, pericentric foci (Figure 6). By contrast, in interphase pMEFs lacking Topors, few cells exhibited pericentric concentration of HP1α, with most cells exhibiting a diffuse nuclear localization of the protein (Figure 6A). Loss of Topors did not affect the nuclear content of HP1α as assessed by immunoblotting (Figure 6B). Since HP1α is required for cohesin recruitment to centromeres [15], mislocalization of HP1α provides an explanation for the aneuploidy observed in Topors-deficient pMEFs.

HP1α localization to pericentric regions has been shown to require both methylation of lysine 9 on histone H3, as well as small interfering RNAs originating from pericentric repetitive DNA [16-18]. Deficiency of Topors did not affect the pericentric enrichment of trimethylated H3K9 in interphase cells, or the overall nuclear content of trimethylated H3K9 as assessed by immunoblotting (Figure 6). Similarly, no difference was observed in the diffuse nuclear distribution of H3K9 acetylation in immunofluorescence studies of interphase pMEFs, or in total H3K9 acetylation in *Topors*^-/- ^versus *Topors*^+/+ ^pMEFs (Additional File 1: Figure S2).

By constrast, studies of RNA transcribed from pericentric (major satellite repeat) DNA in pMEFs demonstrated increased amounts of these transcripts in *Topors*^-/- ^pMEFs compared to *Topors*^+/+ ^pMEFs (Figure 6C). These results suggest that the altered HP1α localization in Topors-deficient pMEFs may relate to an alteration in pericentric repetitive DNA transcription.

Since Topors is capable of functioning as both a ubiquitin and SUMO E3 ligase, the alterations in HP1α localization and pericentric DNA transcription in Topors-deficient pMEFs may relate to loss of ubiquitination and/or sumoylation of a centromeric Topors substrate. While further work is needed to identify Topors substrates, we investigated whether deficiency of Topors affected total levels of ubiquitin, SUMO-1, or SUMO-2/3 conjugates in pMEFs. No differences in the pattern of ubiquitin or SUMO-1 conjugates were observed in *Topors*^-/- ^pMEFs compared to *Topors*^+/+ ^pMEFs (Figure 6D). In addition, the pattern of low molecular weight SUMO-2/3 conjugates was similar in *Topors*^-/- ^and *Topors*^+/+ ^pMEFs (Figure 6D). However, a small, but reproducible decrease was suggested in high molecular weight SUMO-2/3 conjugates in *Topors*^-/- ^pMEFs compared to *Topors*^+/+ ^pMEFs (Figure 6D). Recently, polymeric conjugation of SUMO-2/3 was implicated in centromeric function [19,20], raising the intriguing possibility that the changes in pericentric heterochromatin observed in Topors-deficient cells may relate to alteration in cellular levels of polymeric SUMO-2/3 conjugates.

### Deficiency of Topors confers a transcriptional state similar to exposure to histone deacetylase inhibitors and results in resistance to trichostatin A

To gain additional insight into the cellular function of Topors, we compared gene-expression profiles for *Topors*^+/+ ^and *Topors*^-/- ^pMEFs. Using the Affymetrix Mouse Genome 430 2.0A array, RNA was analyzed from three independent cultures of each cell type. Selecting a false discovery rate of 0.0001, 152 probe sets were identified as differentially expressed in *Topors*^+/+ ^versus *Topors*^-/- ^cells. Among these 152 probe sets, 88 had at least a mean 3-fold alteration in transcript level, representing 73 genes: 48 upregulated and 25 downregulated in the *Topors*^-/- ^MEFs (Additional File 1: Table S1). Topors was identified as the most highly downregulated transcript in *Topors*^-/- ^MEFs (Additional File 1: Table S1). We confirmed differential expression of 11 of the 73 genes using semi-quantitative RT-PCR (Additional File 1: Figure S3).

**Figure 7:**
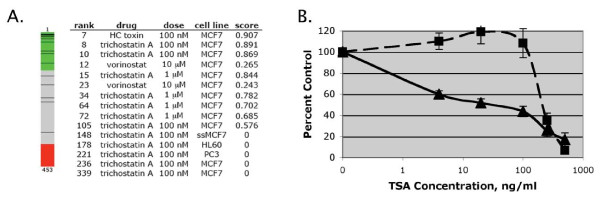
**Loss of Topors confers a gene expression profile similar to histone deacetylase inhibition and resistance to trichostatin A**. **A**. From a collection of 453 gene expression profiles representing 164 compounds, the expression profiles of the histone deacetylase inhibitors HC toxin, trichostatin A, and vorinostat are highly similar to the gene expression profile of murine embryonic fibroblasts lacking Topors. The barview (left) is constructed from 453 horizontal lines, each representing an individual treatment instance and ordered by their similarity to the Topors signature. All HC toxin (n = 1), trichostatin (n = 12), and vorinostat (n = 2) instances are shown as black lines. Colors applied to the remaining instances reflect positive (green), negative (red), or no (gray) similarity to the Topors signature. The table shows the rank, dose, cell line, and connectivity score for each histone deacetylase inhibitor treatment instance. **B**. *Topors*^+/+ ^(solid lines) and *Topors*^-/- ^(dotted lines) MEFs were incubated for 72 hours with increasing concentrations of trichostatin A. Anti-proliferative activity was determined using an MTT assay. Data represent means and standard deviations of 3-6 replicates. Estimated IC_50 _values for trichostatin A are 30 ng/ml for *Topors*^+/+ ^cells versus 232 ng/ml for *Topors*^-/- ^cells (P < 0.0001, F test).

Many of the differentially expressed genes are implicated in tumorigenesis, and 5 of the upregulated genes are involved in the WNT pathway (*e.g. Fst, Lgr5, Sfrp1, Sfrp2, and Wisp2*, Additional File 1: Table S1), which is dysregulated in a variety of cancers [21]. This finding is similar to a report implicating Topors (TP53BPL) in the transforming growth factor β signaling pathway [22]. In addition, among the 73 differentially expressed genes, 57 (40 upregulated, 17 downregulated) had identifiable human orthologs that allowed comparison to 453 gene expression profiles generated by treatment of human cell lines with 164 different small molecules: the "Connectivity Map" [23]. The gene expression profile associated with deficiency of Topors showed strong similarity to the gene expression profiles associated with three different histone deacetylase inhibitors: HC toxin, trichostatin A, and vorinostat (Figure 7, see Additional File 1: Table S2 for complete results). The similarity of the Topors deficiency signature to histone deacetylase inhibition is supported by the occurrence of several replicates with high connectivity scores, and by the finding that structurally distinct HDAC inhibitors show this similarity (Figure 7). No other class of compounds was similarly represented among the gene expression profiles most highly ranked with the Topors profile (Additional File 1: Table S2). In addition, when evaluating permuted results, which estimate the enrichment of specific compounds among the most highly ranked profiles, both vorinostat and trichostatin A were associated with low permutation p-values (0.0058 and 0.0363, respectively). Together, these findings suggest that loss of Topors generates a cellular state that is similar to that created by exposure of cells to histone deacetylase inhibitors.

To further explore the finding that loss of Topors conferred a cellular state similar to that of histone deacetylase inhibition, we investigated the sensitivity of Topors-deficient pMEFs to the histone deacetylase inhibitor trichostatin A. The results indicate that loss of Topors confers a high level of resistance to the anti-proliferative effects of this compound (Figure 7). This characteristic is not associated with general resistance to anti-proliferative drugs, since *Topors*^-/- ^pMEFs are not resistant to DNA-damaging agents or to the microtubule-targeting drug vinblastine (Figure 5).

## Discussion

Our studies indicate that mice lacking the dual ubiquitin and SUMO E3 ligase Topors are viable, but exhibit increased perinatal mortality and decreased weight, as well as decreased lifespan. Since there are several ubiquitin and SUMO E3 ligases in mammals [24], it is not surprising that Topors is dispensable for viability. Our finding that steady state amounts of total cellular ubiquitin and SUMO-1 conjugates in pMEFs are unaffected by loss of Topors is also consistent with redundancy of ubiquitin and SUMO E3 ligases. However, the apparent decrease in high molecular weight SUMO-2/3 conjugates in Topors-deficient pMEFs suggests that Topors may have a non-redundant role in regulation of polymeric SUMO-2/3 chains. We note that a similar specific role for Topors in polymeric (as opposed to monomeric) SUMO conjugation was identified in studies of Topors-induced sumoylation of topoisomerase I, although these studies employed SUMO-1 transfections and did not examine SUMO-2/3 [4].

Similar to our results, an increase in perinatal mortality as well as decreased weight were reported in mice lacking the SUMO E3 ligase PIAS1 [25], although total SUMO-1 and SUMO-2/3 conjugates were unaffected in thymocytes from PIAS1-deficient mice [25]. A decrease in the expected number of homozygotes from heterozygote matings was observed in mice lacking the related SUMO E3 ligase PIASy, although this was strain dependent, and mice lacking PIASy were phenotypically normal [26]. Loss of PIASy did not affect total SUMO-1 or SUMO-2/3 conjugates in MEFs [26]. Loss of the SUMO E3 ligase RANBP2 results in embryonic lethality [27]. The effect of RANBP2 deficiency on cellular SUMO conjugates was not reported in this study [27]. Analogous to mice lacking PIAS1, there were no gross organ defects in mice homozygous for the mutant *Topors *allele, and additional studies will be necessary to determine the etiology of both the perinatal mortality as well as the decreased lifespan observed in Topors-deficient mice.

Our finding that deficiency of Topors results in an increased rate of malignancy in mice supports previous analyses implicating *TOPORS *as a tumor suppressor in various human malignancies [5,1,6]. Moreover, our studies of primary embryonic fibroblasts provide the first evidence that loss of Topors confers genetic instability. Specifically, complete loss of Topors results in a high rate of aneuploidy, which is due to a defect in mitotic chromosome segregation, rather than a defect in DNA repair. Similar to other tumor suppressor genes involved in chromosome segregation rather than DNA repair, malignancies in Topors-deficient mice occurred predominantly in older mice, and in a variety of tissues [28]. Notably, the finding that *Topors*^+/- ^pMEFs did not exhibit an increased rate of aneuploidy suggests that additional events, such as alteration of the remaining wild-type allele, underly the increase in tumorigenesis observed in *Topors*^+/- ^mice.

Studies of the mechanisms underlying the chromosome segregation defect in Topors-deficient pMEFs indicated that loss of Topors did not affect the mitotic checkpoint, but resulted in altered pericentric heterochromatin, manisfested by mislocalization of pericentic HP1α as well as increased transcription from pericentric major satellite repetitive DNA. This finding suggests a role for Topors in pericentric heterochromatin maintenance, and adds to several lines of evidence implicating Topors in chromatin regulation. First, Topors co-localizes with PML nuclear bodies in human cells [7]. Among other functions, PML nuclear bodies were shown to contain pericentric satellite DNA and HP1α, and were specifically implicated in establishment of pericentric heterochromatin [29]. Similarly, although the human antibody was not able to recognize murine Topors in immunohistochemical studies, cell fractionation studies of pMEFs indicated that the majority of the Topors protein was present in a fraction containing chromatin and nuclear matrix components ([30], see Methods).

Second, a *Drosophila Topors (dTopors) *ortholog interacts with Mod(mdg4)2.2, a member of the *gypsy *transposon chromatin insulator complex [11]. Chromatin insulators are DNA sequences defined according to an ability to both block enhancer-promoter communication, and to shield genes from silencing conferred by nearby chromatin modifications such as histone deacetylation [31]. In genetic studies, *dTopors *was shown to be required for *gypsy *insulator function, and the dTopors protein was shown to co-localize with nuclear lamin [11]. In mammals, insulators are important in genomic imprinting, which results in expression of only the maternal or paternal allele [32]. Notably, among the 73 differentially expressed genes identified in our transcriptional profiling analysis of Topors-deficient pMEFs, 10 were identified as being imprinted by an expression profiling screen [33] (Additional File 1: Table S1). Among these, *Xist *and *Peg3 *are well-known imprinted genes [34,35]. Notably, the transcription of other imprinted genes, such as *Igf2 *and *U2af1-rs1 *(probe sets for both are present on the Affymetrix Mouse Genome 430A 2.0 array) was not identified as altered in *Topors*^-/- ^pMEFs, indicating that loss of Topors does not result in global deregulation of imprinting.

Third, a search for Topors sumoylation substrates using a proteomic in vitro screen yielded several chromatin-related proteins [10]. Among these potential substrates, KRAB-associated protein 1 (KAP-1)/transcription intermediary factor 1-beta (TIF1-β), histone H4, and Ku70 are known to interact with HP-1 proteins [14]. In addition, the p48 subunit of chromatin assembly factor 1 (CAF1) was identified as a putative Topors substrate [10], with CAF1 implicated in recruitment of HP-1α to pericentric heterochromatin [36]. Furthermore, mSin3A was identified as a potential Topors sumoylation substrate, and transfection studies indicated that Topors is capable of sumoylating mSin3A in cells [10]. Since alterations in mSin3A function lead to chromosomal and genomic instability and accelerate tumorigenesis [37,38], it is possible that loss of sumoylation or ubiquitination of mSin3A or other chromatin proteins by Topors is important in the altered pericentric heterochromatin observed in Topors-deficient MEFs.

In this regard, there are several examples of heterochromatin and chromosome segregation defects conferred by alterations in components of ubiquitination or sumoylation pathways in eukaryotes. Deficiency of the ubiquitin E3 ligase BRCA1 results in chromosomal instability [39,40], as well as defects in the chromatin structure of X chromosomes [41]. BRCA1 localizes in pericentric heterochromatin regions in MEFs [42] and was also identified as a member of a SWI/SNF chromatin remodeling complex [43]. In addition, a ubiquitin E3 ligase complex containing cullin 4 was shown to be required for heterochromatin formation and proper chromosomal segregation in fission yeast [44-46]. Interestingly, this E3 ligase was also shown to regulate transcription of centromeric repetitive DNA in yeast [44].

Similarly, deficiency of the SUMO-conjugating enzyme Hus5/Ubc9 results in abnormal chromosome segregation in fission yeast [47], and in loss of gene silencing as well as altered histone modification patterns at heterochromatic regions [48]. Murine cells lacking the SUMO E2 enzyme Ubc9 exhibit mitotic chromosome defects, including anaphase bridges [49]. Knockdown of the SUMO E3 ligase PIASγ in human HeLa cancer cells results in a defect in mitotic chromosome segregation, possibly due to loss of topoisomerase II sumoylation [50]. Loss of Pli1p, a PIAS ortholog in fission yeast, also results in chromosomal instability and reduced transcriptional silencing of centromeric DNA [51].

Although further studies are needed to determine if the ubiquitination or sumoylation activities of Topors are required for maintenance of pericentric heterochromatin, our finding that loss of Topors may result in a decrease in polymeric SUMO-2/3 conjugates adds to increasing evidence implicating polymeric SUMO-2/3 chains in centromere function. For example, polymeric SUMO-2/3 conjugates are implicated in localization of topoisomerase IIα to centromeres [52,19], and are required for localization of CENP-E to kinetochores [20].

Our studies of Topors-deficient cells are also relevant to the recent discovery of histone deacetylases as therapeutic targets in cancer [53]. Loss of Topors results in resistance to the anti-proliferative effects of histone deacetylase inhibitors, without affecting cellular sensitivity to DNA-damaging or microtubule-targeting agents. Interestingly, aneuploidy, mislocalization of HP1α, as well as decreased silencing of pericentric DNA [54], occur in cells treated with histone deacetylase inhibitors [55-57].

## Conclusion

Our studies indicate that mice lacking the dual ubiquitin and SUMO E3 ligase Topors are viable, but exhibit increased perinatal mortality and decreased weight, as well as decreased life span. Complete loss of Topors results in a high rate of aneuploidy in primary embryonic fibroblasts, which is due to a defect in mitotic chromosome segregation, rather than a defect in DNA repair. Studies of the mechanisms underlying the chromosome segregation defect in Topors-deficient pMEFs indicated that loss of Topors did not affect the mitotic checkpoint, but resulted in altered pericentric heterochromatin, manifested by mislocalization of pericentic HP1α as well as increased transcription from pericentric major satellite repetitive DNA. These results indicate a unique role for Topors in maintenance of genomic stability and pericentric heterochromatin. Loss of Topors also resulted in resistance to the anti-proliferative effects of histone deacetylase inhibitors. Since relatively little is known regarding mechanisms of resistance to histone deacetylase inhibitors, elucidation of the mechanisms by which loss of Topors confers resistance to these drugs may be useful in efforts to maximize their effectiveness in the treatment of patients with cancer.

## Methods

All animal studies were approved by the University of Medicine and Dentistry of New Jersey Institutional Animal Care and Usage Committee (IACUC). The IACUC approval number is I06-046.

### Generation and genotyping of Topors-deficient mice and embryonic fibroblasts

129/OlaHsd embryonic stem (ES) cells containing a gene-trapped *Topors *allele were obtained from BayGenomics (DTM034, http://baygenomics.ucsf.edu/). RNA and DNA sequencing indicated that in these cells, the *Topors *locus is interrupted by insertion of the pGT1dTMpfs vector into *Topors *intron 2 (http://baygenomics.ucsf.edu and data not shown). Blastocyst injections were performed with DTM034 ES cells and a chimeric 129/OlaHsd/C57BL/6J male was obtained. Germline transmission of the mutant allele was achieved in a C57BL/6J background, and all mice described in this study were maintained in a hybrid 129/OlaHsd and C57BL/6J background. Mice were genotyped by analyses of DNA obtained from tail clippings, using specific PCR and primers hybridizing to sequences in *Topors *intron 2 and the pGT1dTMpfs vector, as described in Additional File 1: Methods. Embryonic fibroblasts were generated from E13.5 embryos using standard methods, and were grown in DMEM media containing 10% fetal bovine serum, 1% L-glutamine and 100 U/ml penicillin and 100 μg/ml streptomycin.

### Topors RNA and protein analyses

Topors RNA expression was analyzed in mouse tissues and primary embryonic fibroblasts by RT-PCR using two sets of primers: one set with the upstream primer designed to hybridize to exon 2 and the downstream primer to exon 3, yielding a 1.2 kb product. For the second primer set, both primers were designed to hybridize to exon 3, yielding a 660 bp product. Primers hybridizing to β-actin were used as a control. Primer sequences and details of the RT-PCR conditions are provided in Additional File 1: Methods.

Topors protein expression was assessed in tissues and embryonic fibroblasts using a chromatin extraction method as described previously [30]. Briefly, cells were resuspended or tissue was homogenized in buffer A (10 mM HEPES, pH 7.9, 10 mM KCl, 1.5 mM MgCl_2_, 0.34 M sucrose, 10% glycerol, 1 mM dithiothreitol, 0.5 μg/ml leupeptin, 1 μg/ml pepstatin, and 1 mM PMSF). Triton X-100 was added to a final concentration of 0.1%. Following incubation on ice, the nuclei (fraction P1) were collected by centrifugation at 1,300 × *g*. The nuclei were washed once in buffer A and resuspended in buffer B (3 mM EDTA, 0.2 mM EGTA, 1 mM DTT with protease inhibitors as described above). Following a 30-minute incubation on ice, soluble (fraction S2) and insoluble (chromatin, fraction P2) fractions were separated by centrifugation for 5 minutes at 1,700 × *g*. The insoluble chromatin pellet was further processed by resuspension in a solution containing 10 mM Tris-Cl, 10 mM KCl, 1 mM CaCl_2_, and 1 U micrococcal nuclease (Sigma). After 5 minutes of incubation at 37°C, the reaction was stopped by addition of 1 mM EGTA. Fractions were analyzed by boiling for 10 minutes in SDS-sample buffer (60 mM Tris-Cl, pH 6.8, 2% SDS, 10% glycerol, and 0.1% phenol red) containing 1 mM DTT, followed by SDS-polyacrylamide electrophoresis and immunoblotting using a polyclonal antibody generated against human Topors [7], as well as a tubulin antibody (Sigma). The majority of the Topors protein in both tissues and embryonic fibroblasts was present in the chromatin fraction P2.

### Embryonic fibroblast growth, cell cycle, and karyotype analyses

Growth rates of murine embryonic fibroblasts generated from E13.5 embryos were determined by seeding 100,000 early passage cells in triplicate. Viable cell counts were determined each day using an automated cell counter (Beckman Vi-Cell Coulter Counter). Cell cycle distributions were determined for exponentially growing cells using ethanol fixation, propidium iodide staining, and flow cytometry as described previously [1].

Cellular foci formation was assessed by seeding 10^6 ^cells in DMEM media in triplicate. After a period of 3 weeks, cells were fixed in methanol and stained with Giemsa for manual counting of foci. Anchorage-independent growth was evaluated by seeding 5 × 10^4 ^cells in DMEM media containing 0.3% low melting point agarose. After 2 weeks the cells were stained with crystal violet and colonies counted manually.

Metaphase chromosome spreads were obtained by treating cells with 0.13 μg/ml colcemid for 2 hours to induce mitotic arrest. Metaphase chromosome spreads were trypsin-Giemsa banded and karyotypes analyzed as described previously [58].

### Drug and ionizing radiation anti-proliferative assays

Topotecan and vinblastine were obtained from GlaxoSmithKline (King of Prussia, PA, USA) and Sigma-Aldrich Chemical Company (St. Louis MO, USA), respectively. Stock solutions were maintained as 10 mM concentrations in DMSO. Radiation experiments were performed using a cesium 137-based cell irradiator (model IBL-473C, CIS-US, Inc). Anti-proliferative effects of drugs and ionizing radiation on primary embryonic fibroblasts were determined using tetrazolium dye-based MTS or MTT assays as described [59]. Concentrations associated with 50% inhibition of growth (IC_50_) were identified using non-linear (sigmoidal) regression analyses (Prism, GraphPad Software, Inc.).

### Immunofluorescence and immunoblotting assays

For immunofluouresence studies, exponentially proliferating cells were grown on cover slips, then fixed in 4% paraformaldehyde for 10 minutes at room temperature. After washing and permeabilization with 0.5% Triton X-100, the cells were incubated for 1 hour at room temperature with the primary antibody diluted in 5% goat serum. Primary antibodies were used that recognized H2A.X phosphorylated on serine 139 (Millipore/Upstate, Bedford, MA), HP-1α, (Millipore/Upstate), trimethyl-histone H3 (Lys9) (Millipore/Upstate), acetyl-histone H3 (Lys9) (Abcam, Cambridge, MA) and α-tubulin (Millipore/Upstate). After washing, the cells were incubated with the appropriate secondary antibody in 5% goat serum. Cells were mounted onto slides using DAPI-containing mounting media (Vectashield, Vector Laboratories, Inc., Burlington, CA).

For immunoblotting studies, nuclear and chromatin proteins were obtained as described above. For analysis of total cellular ubiquitin, SUMO-1, and SUMO-2/3 conjugates, cells were lysed in SDS-sample buffer containing 25 mM N-ethylmaleimide (Sigma). In addition to the antibodies listed for immunofluorescence studies, immunoblotting antibodies included total histone H3 (Sigma), α-tubulin (Sigma), ubiquitin (Santa-Cruz), SUMO-1 (Zymed), and SUMO-2/3 (Zymed).

### Pericentric major satellite transcript analyses

These assays were performed in a manner similar to that described by Lehnertz, et al. [60]. Briefly, total RNA was extracted using RNeasy (Qiagen), then treated with TURBO DNase (Ambion). First strand cDNA was synthesized in a 20 μl volume using 1 μg of RNA, 200 U Superscript II reverse transcriptase (Invitrogen), 0.5 μg/μl random hexadeoxynucleotide primers (Promega), and 0.5 mM dNTPs. 2 μl of the cDNA product (or a 10-fold dilution of this product) was used in a PCR reaction containing1 nM of major satellite primers (forward, 5'-GACGACTTGAAAAATGACGAAATC; reverse, 5'-CATATTCCAGGTCCTTCAGTGTGC), or GAPDH primers (forward, 5'-AACAACCCCTTCATTGACCTC; reverse, 5'-TTCTGAGTGGCAGTGATGGC). Reactions also contained 1.5 mM MgCl_2_, 50 mM KCl, 20 mM Tris-HCL (pH 8.4), 0.2 mM dNTPs, and 2 units Taq DNA polymerase (Invitrogen). Thermocycling parameters for the PCR reactions were as follows: 95°C for 2 minutes, followed by 35 cycles of 94°C, 30 seconds/60°C, 45 seconds/72°C, 90 seconds, followed by a final incubation at 72°C for 10 minutes. PCR products were analyzed by 1.5% agarose gel electrophoresis and ethidium bromide staining.

### Gene expression profiling and analyses

Transcriptional profiling was performed utilizing 15 μg total RNA obtained from 3 independent cultures of *Topors*^+/+ ^and *Topors*^-/- ^E13.5 early passage embyronic fibroblasts. RNA was isolated (RNeasy, Qiagen) and reverse transcribed using oligo-dT primers. Following RNase H-mediated second-strand cDNA synthesis, double-stranded cDNA was purified and transcribed using T7 RNA Polymerase and a biotinylated nucleotide analog/ribonucleotide mix (One-Cycle Target Labeling and Control Reagents, Affymetrix). The resulting biotinylated cRNA was hybridized to an Affymetrix Mouse Genome 430A 2.0 microarray. Gene expression values were determined using GCOS software (version 1.4, Affymetrix) and a global scaling normalization method (target value 150). The data were log-transformed and analyzed using a modified t-test and a defined false discovery rate as described by Tusher and Efron [61,62].

The Topors deficiency signature was developed by selecting genes that were associated with a false discovery rate of 0.0001, and for which there was at least a 3-fold mean difference in transcript expression. Among the 73 genes that met these criteria, 57 had identifiable human orthologs and thus comprised the Topors deficiency signature. This signature was compared to 453 gene expression profiles present in the Connectivity Map [23]. For each of the 453 profiles, a connectivity score was calculated (based on the Kolmogorov-Smirnov statistic), which represents the relative similarity of the profile to the Topors deficiency signature.

## Authors' contributions

HM performed most of the experiments. MB participated in the generation of Topors-deficient mice. HA performed the pathological evaluations. DM carried out all the statistical evaluations for the study. MY performed immunoflourescence experiments. JD helped in the validation of the Topors antibody in mouse lysates. HR performed most of the mouse genotyping experiments. MG performed necropsy and dissection in mice. SG designed and coordinated the immunoflourescence and karyotype studies. AS performed all of the western blotting as well as mouse colony maintenance and breeding. ER conceived the study, coordinated in the design and drafted the manuscript. All authors read and approved the final manuscript.

## Supplementary Material

Additional file 1 **Additional figures, tables, and methods**. Figure S1. Representative SKY karyotype of a *Topors*^-/- ^pMEF cell, containing 79 chromosomes. Figure S2. Immunofluorescence and immunoblotting studies of H3K9 acetylation in *Topors*^+/+ ^and *Topors*^-/- ^pMEFs. Figure S3. Semi-quantitative RT-PCR analyses of selected genes identified as differentially expressed in *Topors*^+/+ ^and *Topors*^-/- ^pMEFs by microarray analyses. Table S1. Differentially Expressed Genes in *Topors*^+/+ ^and *Topors*^-/- ^primary murine embryonic fibroblasts. Table S2. Connectivity Map Results for the Topors Deficiency Signature. Methods: Genotyping methods, RT-PCR methods Click here for file
