# Supplementary material for: Deficiency of the dual ubiquitin/SUMO ligase Topors results in genetic instability and an increased rate of malignancy in mice
Source: BMC Mol Biol. 2010 Apr 29;11:31. doi: 10.1186/1471-2199-11-31 (PMC2873312; doi:10.1186/1471-2199-11-31)
Supplement: Additional file 1 — Additional figures, tables, and methods. Figure S1. Representative SKY karyotype of a Topors-/- pMEF cell, containing 79 chromosomes. Figure S2. Immunofluorescence and immunoblotting studies of H3K9 acetylation in Topors+/+ and Topors-/- pMEFs. Figure S3. Semi-quantitative RT-PCR analyses of selected genes identified as differentially expressed in Topors+/+ and Topors-/- pMEFs by microarray analyses. Table S1. Differentially Expressed Genes in Topors+/+ and Topors-/- primary murine embryonic fibroblasts. Table S2. Connectivity Map Results for the Topors Deficiency Signature. Methods: Genotyping methods, RT-PCR methods [file 1471-2199-11-31-S1.DOC]

**Methods:**

Genotyping by PCR. DNA was obtained from tail clippings or embryonic fibroblasts using the DNeasy kit (Qiagen). For detection of the mutant *Topors* allele, PCR was performed using Advantage 2 DNA polymerase (Clontech) and an upstream primer hybridizing to sequences in the pGT1dTMpfs vector (P1, 5'-TGGAACGAAAACTCACGTTAAGGG), with a downstream primer hybridizing to sequences in *Topors* intron 2 (P2, 5'-CCCTACAGCTATATGGATATAAGCAGC). The resulting PCR product is 800 bp. For detection of the wild-type allele, primer P2 was used with an upstream primer hybridizing to sequences in *Topors* intron 2 (P3, 5'-CCTGGGTCAAGCATTTTC). The resulting PCR product is 1 kb. Thermocycling parameters for both PCRs were as follows: 94°C for 5 minutes, then 10 cycles of 94°C, 30 seconds/75°C to 70°C, 30 seconds (with a decrease in temperature by 1°C every 2nd cycle, then 35 cycles of 94°C, 30 seconds/65°C, 30 seconds/72°C, 60 seconds, followed by a final incubation at 72°C for 8 minutes. PCR products were analyzed by 1.5% agarose gel electrophoresis and ethidium bromide staining.

Topors and Actin RT-PCR assays. RNA was obtained from mouse tissues and embryonic fibroblasts using the RNeasy kit (Qiagen). Purified RNA was subsequently treated with Turbo DNase (Ambion) before use in RT-PCR assays. Reverse transcription and PCR were performed in a single tube (Qiagen OneStep RT-PCR Kit). For detection of Topors mRNA, RT-PCR was performed using a downstream primer hybridizing to sequences in *Topors* exon 3 (5'-CCAGGAGTTTCATCATCCCACGG), with upstream primers hybridizing to sequences in *Topors* exon 2 (5'-CTCGCGAGGAGGGTGAAG, yielding a 1.2 kb product) or to sequences in exon 3 (5'-GGAGAACAACCACGCCACCAGATAG, yielding a 660 bp product). For β-actin RT-PCR, the upstream primer sequence was 5'-GTGGGCCGCTCTAGGCACCAA, with the downstream primer sequence 5'-CTCTTTGATGTCACGCACGATTTC). Thermocycling parameters for RT-PCR reactions were as follows: 50°C for 45 minutes, then 95°C for 15 minutes, followed by 35 cycles of 94°C, 30 seconds/60°C, 45 seconds, 72°C, 60 seconds, followed by a final incubation at 72°C for 10 minutes. PCR products were analyzed by 1.5% agarose gel electrophoresis and ethidium bromide staining.


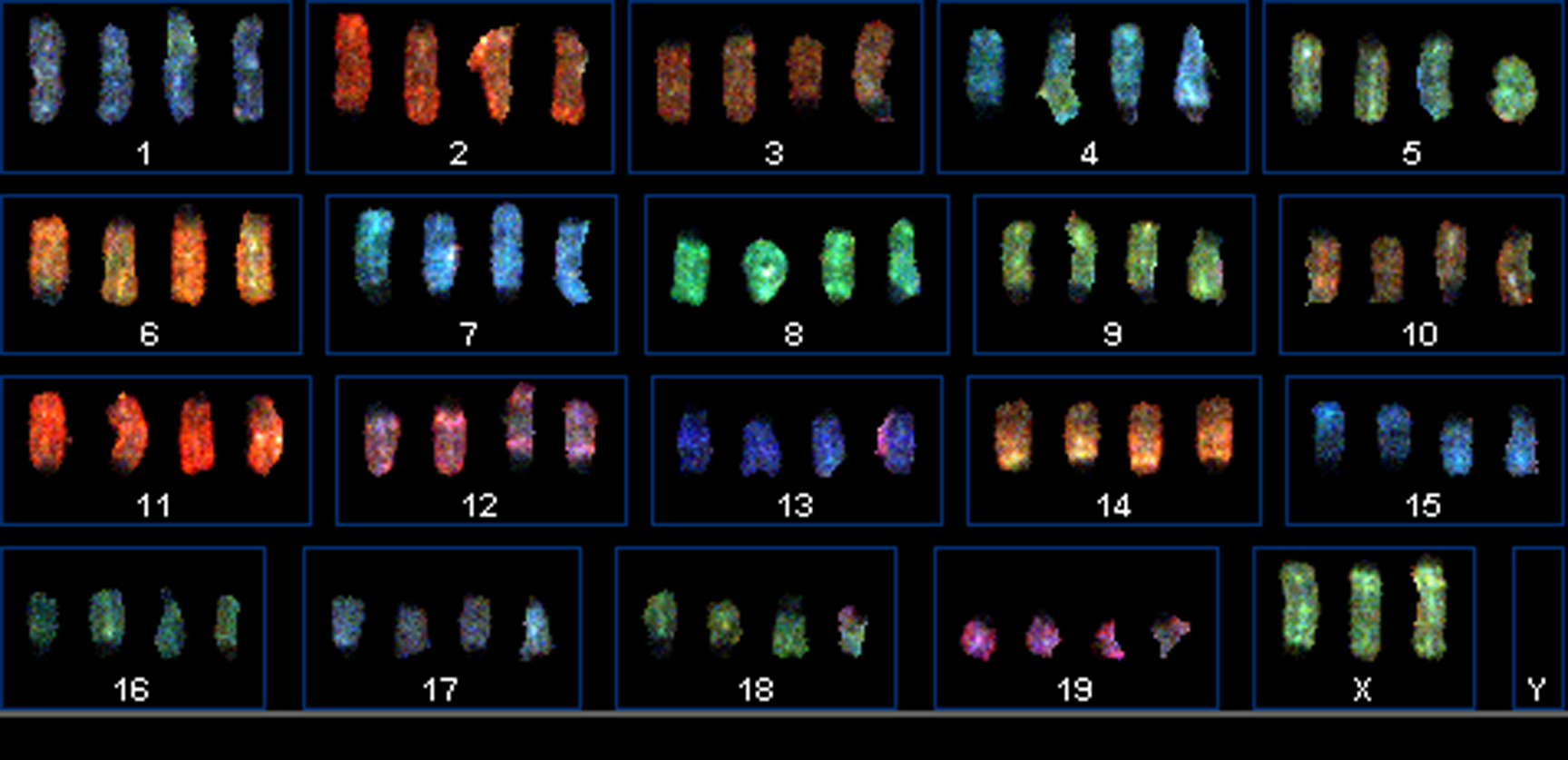


**Figure 1.** Representative SKY karyotype of a *Topors^-/-^* pMEF cell, containing 79 chromosomes. The SKY analysis was performed by the Roswell Park SKY facility using previously described methods [Matsui, 2002 #346].


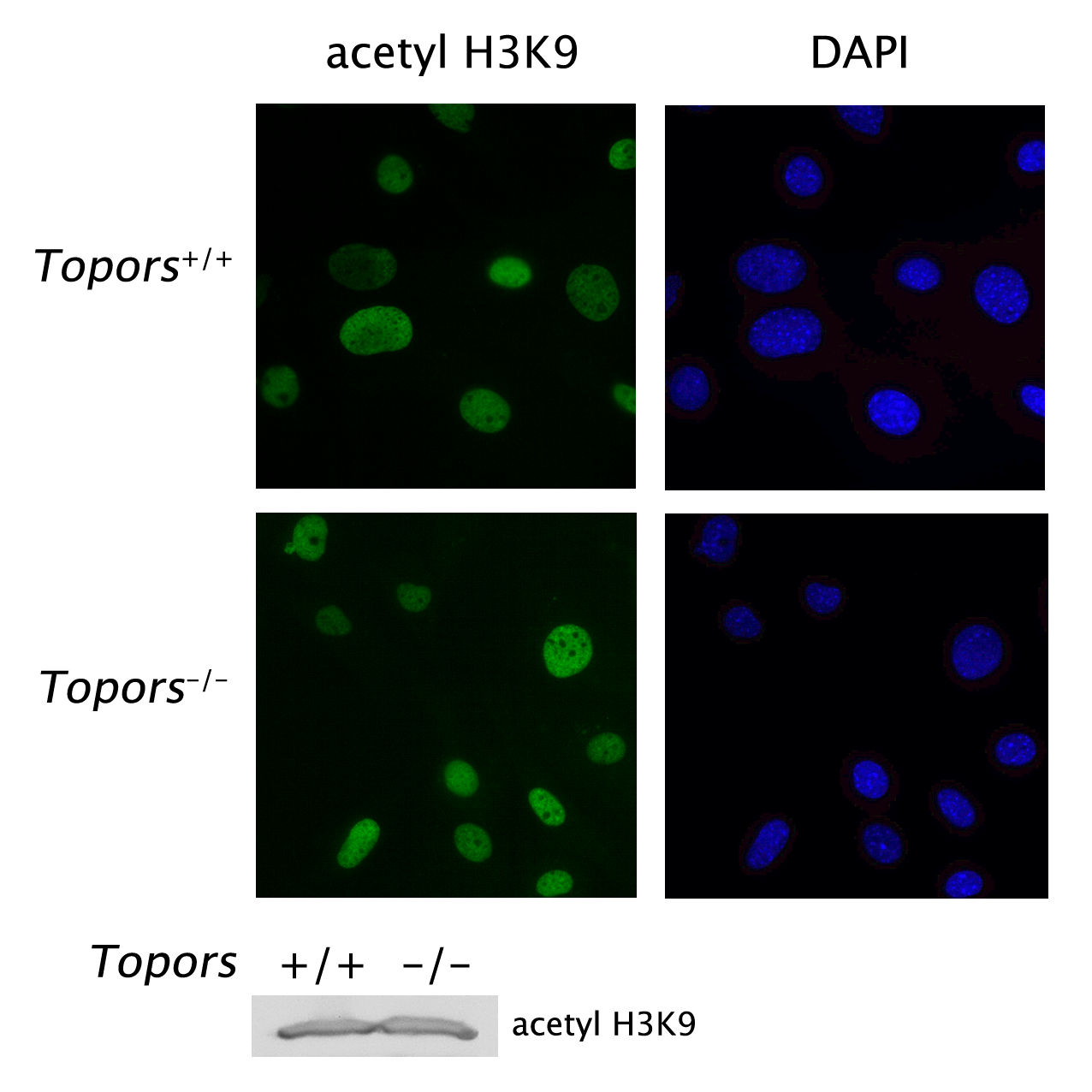


**Figure 2. Immunofluorescence and immunoblotting studies of H3K9 acetylation in *Topors^+/+^* and *Topors^-/-^* pMEFs.** Shown are representative immunofluorescence images of *Topors^+/+^* and *Topors^-/-^* pMEFs using an antibody recognizing acetylated histone H3K9. The lower image shows the results of immunoblotting of nuclear lysates with the acetylated H3K9 antibody. *Topors^+/+^* and *Topors^-/-^* pMEF nuclear lysates exhibited similar amounts of total histone H3, as shown in Figure 8.


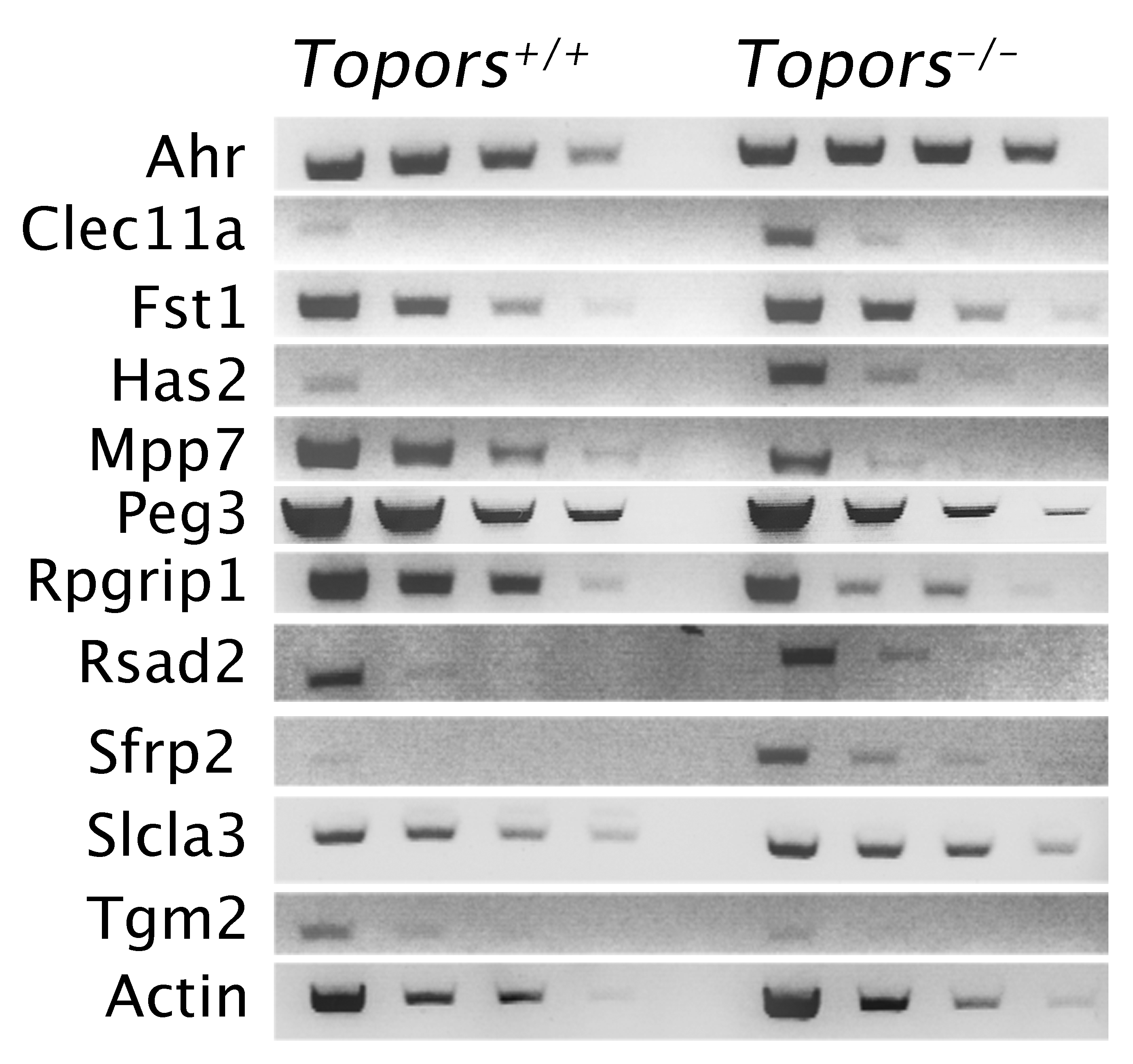


**Figure 3. Semi-quantitative RT-PCR analyses of selected genes identified as differentially expressed in *Topors^+/+^* and *Topors^-/-^* pMEFs by microarray analyses.** RNA extracted from *Topors^+/+^* and *Topors^-/-^* pMEFs was subjected to semi-quantitative RT-PCR using primers specific for the indicated genes. Lanes from left to right represent serial 3-fold dilutions of total RNA. PCR products were visualized using agarose gel electrophoresis, followed by ethidium bromide staining.

**Table 1. Differentially Expressed Genes in *Topors^+/+^* and *Topors^-/-^* primary murine embryonic fibroblasts**

| **probe set** | **Mean Fold Change in Topors-deficient pMEFs** | **Description (bold indicates human ortholog identified and used for Connectivity Map analysis; genes identified as imprinted [Nikaido, 2003 #236] are underlined)** | **Unigene No.** |
| --- | --- | --- | --- |
| **Upregulated expression** | |  |  |
| 1427300_at | 43.5 | LHX8 - Lim homeobox 8 | Mm.15530 |
| 1419100_at | 36.93 | **Serpina3n** | Mm.22650 |
| 1416776_at | 15.08 | **crystallin, mu;Crym** | Mm.1228 |
| 1424233_at | 12.19 | **mesenchyme homeobox 2** | Mm.341398 |
| 1419379_x_at | 11.77 | **Fxyd2 - FXYD domain-containing ion transport regulator 2** | Mm.22742 |
| 1421421_at | 10.72 | angiopoietin-like 1 (Angptl1) | Mm.389206 |
| 1448201_at | 9.96 | secreted frizzled-related sequence protein 2 (Sfrp2) | Mm.19155 |
| 1436058_at | 9.17 | **radical S-adenosyl methionine domain containing 2 (Rsad2; VHSV induced gene 1; Vig-1)** | Mm.24045 |
| 1427170_at | 9 | Psma8; proteasome (prosome, macropain) subunit, alpha type, 8 | Mm.87277 |
| 1419960_at | 8.37 | Eso-1 (Cphx) | Mm.441404 |
| 1417979_at | 8.06 | **tenomodulin** | Mm.46221 |
| 1422606_at | 7.61 | **C1q and tumor necrosis factor related protein 3 (C1qtnf3)** | Mm.280158 |
| 1427575_at | 7.5 | Fbxw14; F-box protein FBX12 | Mm.296059 |
| 1424234_s_at | 6.98 | **mesenchyme homeobox 2** | Mm.341398 |
| 1448755_at | 6.87 | **Col15a1 procollagen, type XV** | Mm.233547 |
| 1424596_s_at | 6.67 | **LIM and cysteine-rich domains 1 (Lmcd1)** | Mm.234441 |
| 1417732_at | 6.12 | **annexin A8 (Anxa8)** | Mm.3267 |
| 1449102_at | 5.84 | **early B-cell factor 2 (Ebf2)** | Mm.319947 |
| 1452031_at | 5.62 | **solute carrier family 1 (glial high affinity glutamate transporter), member 3 ( Slc1a3)** | Mm.204834 |
| 1418494_at | 5.36 | **early B-cell factor 2 (Ebf2)** | Mm.319947 |
| 1450988_at | 4.93 | **Lgr5 - leucine rich repeat containing G protein coupled receptor 5** | Mm.42103 |
| 1427919_at | 4.84 | **Srpx2 sushi-repeat-containing protein, X-linked 2** | Mm.263553 |
| 1449957_at | 4.6 | Ptprv protein tyrosine phosphatase, receptor type, V | Mm.439765 |
| 1418678_at | 4.42 | **Has2 hyaluronan synthase 2** | Mm.5148 |
| 1419015_at | 4.24 | **WNT1 inducible signaling pathway protein 2 (Wisp2)** | Mm.13828 |
| 1449169_at | 4.23 | **Has2 hyaluronan synthase 2** | Mm.5148 |
| 1421365_at | 4.18 | **Fst - follistatin** | Mm.4913 |
| 1418796_at | 3.98 | **stem cell growth factor (Scgf); Clec11a C-type lectin domain family 11, member a** | Mm.20428 |
| 1426439_at | 3.97 | **DBY RNA helicase (DDX3Y)** | Mm.302938 |
| 1452077_at | 3.91 | **DBY RNA helicase (DDX3Y)** | Mm.302938 |
| 1418632_at | 3.86 | **ubiquitin-conjugating enzyme E2H (Ube2h)** | Mm.332967 |
| 1417210_at | 3.81 | **eukaryotic translation initiation factor 2, subunit 3, structural gene Y-linked (Eif2s3y)** | Mm.250909 |
| 1426438_at | 3.69 | **DBY RNA helicase (DDX3Y)** | Mm.302938 |
| 1427038_at | 3.67 | **preproenkephalin 1; Penk1** | Mm.2899 |
| 1449082_at | 3.5 | **Mfap5; microfibrillar associated protein 5** | Mm.41931 |
| 1423259_at | 3.42 | **Id4; inhibitor of DNA binding 4** | Mm.458006 |
| 1418454_at | 3.34 | **Mfap5; microfibrillar associated protein 5** | Mm.41931 |
| 1460187_at | 3.32 | **Sfrp1; secreted frizzled-related sequence protein 1** | Mm.281691 |
| 1417399_at | 3.27 | **Gas6; growth arrest specific 6** | Mm.3982 |
| 1450171_x_at | 3.1 | **Gzme; granzyme E** | Mm.14424 |
| 1449984_at | 3.1 | **Scyb2, Cxcl2; small inducible cytokine subfamily, member 2** | Mm.4979 |
| 1420855_at | 3.04 | **Eln; elastin** | Mm.275320 |
| 1419728_at | 2.99 | **Scyb5, Cxcl5; small inducible cytokine B subfamily, member 5** | Mm.4660 |
| 1450928_at | 2.98 | **Id4; inhibitor of DNA binding 4** | Mm.458006 |
| 1426598_at | 2.97 | **Uty; ubiquitously transcribed tetratricopeptide repeat gene** | Mm.20477 |
| 1448326_a_at | 2.94 | **Crabp1; cellular retinoic acid binding protein I** | Mm.34797 |
| 1424542_at | 2.88 | **S100a4; pEL98 protein** | Mm.3925 |
| 1416326_at | 2.84 | Crip1; cysteine rich intestinal protein | Mm.272368 |
| 1419598_at | 2.84 | **MS4A6D protein; membrane-spanning 4-domains, subfamily A, member 6D** | Mm.290390 |
| 1456084_x_at | 2.78 | **fibromodulin; Fmod** | Mm.287146 |
| 1424713_at | 2.72 | **Calml4; calmodulin-related protein** | Mm.440576 |
| 1416594_at | 2.71 | **Sfrp1; secreted frizzled-related sequence protein 1** | Mm.281691 |
| 1424754_at | 2.68 | Ms4a7; membrane-spanning 4-domains, subfamily A, member 7 | Mm.193094 |
| 1422631_at | 2.62 | **Ahr; aryl-hydrocarbon receptor** | Mm.30155 |
| 1420731_a_at | 2.62 | **Csrp2; cysteine-rich protein 2** | Mm.2020 |
| 1415904_at | 2.59 | **Lpl; lipoprotein lipase** | Mm.1514 |
| 1449334_at | 2.51 | **Timp3; Similar to tissue inhibitor of metalloproteinase3** | Mm.4871 |
|  |  |  |  |
| **Downregulated expression** | |  |  |
| 1438251_x_at | 2.53 | **HtrA1; HtrA serine peptidase 1; Prss11; protease, serine, 11 (Igf binding)** | Mm.30156 |
| 1421144_at | 2.63 | **Rpgrip1;retinitis pigmentosa GTPase regulator interacting protein 1** | Mm.21662 |
| 1423071_x_at | 2.64 | 6720475J19Rik; gb:AW549928 |  |
| 1448649_at | 2.7 | **Enpep; glutamyl aminopeptidase** | Mm.1193 |
| 1426952_at | 2.76 | Arhgap18; Rho GTPase activating protein 18 | Mm.356496 |
| 1434195_at | 2.78 | **Ube2l6 (ubiquitin-conjugating enzyme E2L 6); Ubce8; ubiquitin-conjugating enzyme 8** | Mm.38261 |
| 1437277_x_at | 2.8 | **Tgm2; transglutaminase 2, C polypeptide** | Mm.330731 |
| 1449979_a_at | 2.82 | **Spock3; sparc/osteonectin, cwcv and kazal-like domains proteoglycan 3** | Mm.334552 |
| 1455179_at | 2.91 | Mpp7; membrane protein, palmitoylated 7 | Mm.133293 |
| 1418876_at | 2.99 | **Foxd1; forkhead box D1** | Mm.347441 |
| 1452180_at | 3.32 | **Phf17; PHD finger protein 17; Jade-1** | Mm.286285 |
| 1417355_at | 3.38 | **Peg3; paternally expressed 3** | Mm.389800 |
| 1419717_at | 3.4 | **Sema3e; sema domain, immunoglobulin domain (Ig), short basic domain, secreted, (semaphorin) 3E; gb:NM_011348.1** |  |
| 1455900_x_at | 3.42 | **Tgm2; transglutaminase 2, C polypeptide** | Mm.330731 |
| 1427797_s_at | 3.48 | **Ctse; cathepsin E** | Mm.230249 |
| 1426936_at | 3.64 | gb:BC002257.1 / IMAGE:3493956 |  |
| 1435106_at | 3.72 | Limch1; LIM and calponin homology domains 1 | Mm.374778 |
| 1419324_at | 3.77 | LIM homeobox protein 9 (Lhx9) |  |
| 1433428_x_at | 3.89 | **Tgm2; transglutaminase 2, C polypeptide** | Mm.330731 |
| 1427605_at | 4.14 | **Hox-2.7; HoxB3; homeo box B3** | Mm.342481 |
| 1423072_at | 4.29 | RIKEN cDNA 6720475J19 gene FL=gb:NM_026586.1; AK020160 |  |
| 1420992_at | 5.1 | **Ankrd1; cardiac responsive adriamycin protein (Crap)** | Mm.10279 |
| 1454906_at | 7.19 | **Top2beta** | Mm.130362 |
| 1420991_at | 7.66 | **Ankrd1; cardiac responsive adriamycin protein (Crap)** | Mm.10279 |
| 1455883_a_at | 10.12 | Lrrtm1 ; leucine rich repeat transmembrane neuronal 1 | Mm.292568 |
| 1421542_at | 10.84 | **DAZ interacting protein 1 (Dzip1)** | Mm.87456 |
| 1427263_at | 20 | **Xist** | Mm.470309 |
| 1416236_a_at | 20 | **Mpzl2; myelin protein zero-like 2** | Mm.33240 |
| 1436936_s_at | 26.17 | **Xist** | Mm.470309 |
| 1417754_at | 31.61 | Topors | Mm.251548 |
| 1417755_at | 41.15 | Topors | Mm.251548 |
| 1427262_at | 95.44 | **Xist** | Mm.470309 |
| 1448838_at | 192.37 | Topors | Mm.251548 |

**Table 2. Connectivity Map Results for the Topors Deficiency Signature**

| rank | instance id | cmap name | batch | dose | cell line | score | up | down |
| --- | --- | --- | --- | --- | --- | --- | --- | --- |
| 1 | 941 | rottlerin | 502 | 10 µM | MCF7 | 1 | 0.328 | -0.222 |
| 2 | 261 | ciclosporin | 20 | 1 µM | MCF7 | 1 | 0.264 | -0.286 |
| 3 | 1070 | troglitazone | 513 | 10 µM | MCF7 | 0.964 | 0.291 | -0.239 |
| 4 | 1053 | prochlorperazine | 513 | 10 µM | MCF7 | 0.929 | 0.361 | -0.15 |
| 5 | 621 | nocodazole | 107 | 1 µM | MCF7 | 0.927 | 0.266 | -0.244 |
| 6 | 165 | exemestane | 7 | 10 nM | MCF7 | 0.922 | 0.24 | -0.267 |
| 7 | 909 | HC toxin | 505 | 100 nM | MCF7 | 0.907 | 0.25 | -0.249 |
| 8 | 1050 | trichostatin A | 513 | 100 nM | MCF7 | 0.891 | 0.271 | -0.219 |
| 9 | 253 | LM-1685 | 18 | 10 µM | MCF7 | 0.878 | 0.334 | -0.149 |
| 10 | 992 | trichostatin A | 506 | 100 nM | MCF7 | 0.869 | 0.371 | -0.107 |
| 11 | 950 | 5211181 | 502 | 12 µM | MCF7 | 0.869 | 0.314 | -0.164 |
| 12 | 1058 | vorinostat | 513 | 10 µM | MCF7 | 0.867 | 0.265 | -0.212 |
| 13 | 983 | haloperidol | 506 | 10 µM | MCF7 | 0.865 | 0.195 | -0.281 |
| 14 | 953 | monorden | 502 | 100 nM | MCF7 | 0.853 | 0.225 | -0.244 |
| 15 | 1014 | trichostatin A | 506 | 1 µM | MCF7 | 0.844 | 0.224 | -0.24 |
| 16 | 252 | celecoxib | 18 | 10 µM | MCF7 | 0.844 | 0.209 | -0.255 |
| 17 | 415 | nordihydroguaiaretic acid | 45 | 1 µM | ssMCF7 | 0.842 | 0.253 | -0.21 |
| 18 | 704 | fulvestrant | 110b | 1 µM | PC3 | 0.835 | 0.188 | -0.271 |
| 19 | 977 | wortmannin | 502 | 1 µM | MCF7 | 0.829 | 0.249 | -0.207 |
| 20 | 1056 | 17-allylamino-geldanamycin | 513 | 1 µM | MCF7 | 0.825 | 0.217 | -0.237 |
| 21 | 578 | 4,5-dianilinophthalimide | 86 | 10 µM | PC3 | 0.82 | 0.176 | -0.275 |
| 22 | 997 | chlorpromazine | 506 | 1 µM | MCF7 | 0.818 | 0.253 | -0.197 |
| 23 | 1000 | vorinostat | 506 | 10 µM | MCF7 | 0.809 | 0.243 | -0.202 |
| 24 | 595 | resveratrol | 95 | 50 µM | MCF7 | 0.807 | 0.24 | -0.204 |
| 25 | 1103 | demecolcine | 514 | 12 µM | MCF7 | 0.807 | 0.161 | -0.283 |
| 26 | 221 | 17-allylamino-geldanamycin | 17 | 100 nM | MCF7 | 0.805 | 0.172 | -0.271 |
| 27 | 1011 | 15-delta prostaglandin J2 | 506 | 10 µM | MCF7 | 0.804 | 0.179 | -0.263 |
| 28 | 1076 | fulvestrant | 513 | 10 nM | MCF7 | 0.802 | 0.189 | -0.252 |
| 29 | 282 | fludrocortisone | 22a | 1 µM | MCF7 | 0.798 | 0.122 | -0.317 |
| 30 | 956 | 5224221 | 502 | 12 µM | MCF7 | 0.789 | 0.247 | -0.187 |
| 31 | 523 | fulvestrant | 74 | 1 µM | ssMCF7 | 0.789 | 0.225 | -0.209 |
| 32 | 826 | prazosin | 504 | 10 µM | MCF7 | 0.785 | 0.261 | -0.171 |
| 33 | 1073 | genistein | 513 | 10 µM | MCF7 | 0.785 | 0.166 | -0.266 |
| 34 | 1072 | trichostatin A | 513 | 1 µM | MCF7 | 0.782 | 0.247 | -0.183 |
| 35 | 990 | alpha-estradiol | 506 | 10 nM | MCF7 | 0.782 | 0.188 | -0.242 |
| 36 | 414 | estradiol | 45 | 10 nM | ssMCF7 | 0.78 | 0.215 | -0.214 |
| 37 | 1006 | 17-allylamino-geldanamycin | 506 | 1 µM | MCF7 | 0.776 | 0.197 | -0.23 |
| 38 | 1057 | monorden | 513 | 100 nM | MCF7 | 0.775 | 0.163 | -0.263 |
| 39 | 1009 | clozapine | 506 | 10 µM | MCF7 | 0.773 | 0.14 | -0.285 |
| 40 | 318 | LY-294002 | 25 | 10 µM | MCF7 | 0.771 | 0.223 | -0.201 |
| 41 | 898 | 5213008 | 505 | 18 µM | MCF7 | 0.771 | 0.186 | -0.238 |
| 42 | 428 | 17-allylamino-geldanamycin | 54 | 1 µM | MCF7 | 0.771 | 0.183 | -0.241 |
| 43 | 1122 | cytochalasin B | 514 | 21 µM | MCF7 | 0.769 | 0.236 | -0.187 |
| 44 | 913 | colforsin | 505 | 50 µM | MCF7 | 0.765 | 0.231 | -0.19 |
| 45 | 222 | tomelukast | 17 | 1 µM | MCF7 | 0.758 | 0.199 | -0.218 |
| 46 | 377 | celecoxib | 39 | 10 µM | MCF7 | 0.756 | 0.19 | -0.226 |
| 47 | 988 | estradiol | 506 | 100 nM | MCF7 | 0.753 | 0.2 | -0.214 |
| 48 | 1068 | thioridazine | 513 | 10 µM | MCF7 | 0.747 | 0.239 | -0.172 |
| 49 | 948 | Y-27632 | 502 | 3 µM | MCF7 | 0.745 | 0.209 | -0.201 |
| 50 | 1077 | LY-294002 | 513 | 10 µM | MCF7 | 0.744 | 0.22 | -0.189 |
| 51 | 384 | tretinoin | 40 | 1 µM | MCF7 | 0.74 | 0.275 | -0.132 |
| 52 | 632 | novobiocin | 109 | 100 µM | SKMEL5 | 0.738 | 0.14 | -0.266 |
| 53 | 368 | pirinixic acid | 36 | 100 µM | MCF7 | 0.735 | 0.181 | -0.223 |
| 54 | 251 | rofecoxib | 18 | 10 µM | MCF7 | 0.724 | 0.207 | -0.191 |
| 55 | 524 | nordihydroguaiaretic acid | 74 | 1 µM | ssMCF7 | 0.72 | 0.222 | -0.174 |
| 56 | 456 | quinpirole | 62 | 1 µM | MCF7 | 0.718 | 0.222 | -0.173 |
| 57 | 1023 | wortmannin | 506 | 10 nM | MCF7 | 0.718 | 0.214 | -0.181 |
| 58 | 433 | valproic acid | 56 | 1 mM | PC3 | 0.718 | 0.165 | -0.23 |
| 59 | 223 | TTNPB | 17 | 100 nM | MCF7 | 0.715 | 0.203 | -0.19 |
| 60 | 958 | resveratrol | 502 | 10 µM | MCF7 | 0.713 | 0.256 | -0.136 |
| 61 | 1060 | valproic acid | 513 | 50 µM | MCF7 | 0.713 | 0.211 | -0.181 |
| 62 | 639 | pentamidine | 111 | 100 µM | MCF7 | 0.709 | 0.157 | -0.233 |
| 63 | 401 | LY-294002 | 42 | 10 µM | ssMCF7 | 0.705 | 0.22 | -0.168 |
| 64 | 873 | trichostatin A | 504 | 1 µM | MCF7 | 0.702 | 0.217 | -0.169 |
| 65 | 610 | monastrol | 101 | 100 µM | MCF7 | 0.702 | 0.206 | -0.18 |
| 66 | 1055 | chlorpromazine | 513 | 1 µM | MCF7 | 0.7 | 0.215 | -0.17 |
| 67 | 999 | monorden | 506 | 100 nM | MCF7 | 0.696 | 0.197 | -0.186 |
| 68 | 949 | 5255229 | 502 | 13 µM | MCF7 | 0.696 | 0.175 | -0.208 |
| 69 | 889 | 5286656 | 505 | 50 µM | MCF7 | 0.691 | 0.158 | -0.222 |
| 70 | 1022 | sirolimus | 506 | 100 nM | MCF7 | 0.691 | 0.155 | -0.225 |
| 71 | 955 | 5248896 | 502 | 11 µM | MCF7 | 0.689 | 0.194 | -0.185 |
| 72 | 981 | trichostatin A | 502 | 1 µM | MCF7 | 0.685 | 0.265 | -0.112 |
| 73 | 262 | indometacin | 20 | 20 µM | MCF7 | 0.685 | 0.242 | -0.135 |
| 74 | 866 | ikarugamycin | 504 | 2 µM | MCF7 | 0.684 | 0.155 | -0.221 |
| 75 | 1003 | nordihydroguaiaretic acid | 506 | 1 µM | MCF7 | 0.682 | 0.184 | -0.191 |
| 76 | 1016 | LY-294002 | 506 | 10 µM | MCF7 | 0.682 | 0.143 | -0.232 |
| 77 | 838 | 5248896 | 504 | 11 µM | MCF7 | 0.676 | 0.135 | -0.237 |
| 78 | 455 | prochlorperazine | 62 | 10 µM | MCF7 | 0.673 | 0.227 | -0.143 |
| 79 | 1069 | 15-delta prostaglandin J2 | 513 | 10 µM | MCF7 | 0.667 | 0.167 | -0.2 |
| 80 | 947 | 17-allylamino-geldanamycin | 502 | 1 µM | MCF7 | 0.667 | 0.157 | -0.21 |
| 81 | 338 | azathioprine | 29 | 100 µM | MCF7 | 0.658 | 0.154 | -0.208 |
| 82 | 416 | clozapine | 46 | 10 µM | MCF7 | 0.653 | 0.178 | -0.181 |
| 83 | 1010 | thioridazine | 506 | 10 µM | MCF7 | 0.651 | 0.242 | -0.116 |
| 84 | 1065 | LY-294002 | 513 | 10 µM | MCF7 | 0.644 | 0.196 | -0.158 |
| 85 | 426 | chlorpromazine | 54 | 1 µM | MCF7 | 0.642 | 0.125 | -0.228 |
| 86 | 984 | acetylsalicylic acid | 506 | 100 µM | MCF7 | 0.629 | 0.157 | -0.189 |
| 87 | 423 | staurosporine | 53 | 100 nM | MCF7 | 0.627 | 0.179 | -0.166 |
| 88 | 893 | pararosaniline | 505 | 10 µM | MCF7 | 0.624 | 0.234 | -0.109 |
| 89 | 378 | tacrolimus | 39 | 1 µM | MCF7 | 0.624 | 0.177 | -0.166 |
| 90 | 335 | nifedipine | 29 | 10 µM | MCF7 | 0.618 | 0.179 | -0.161 |
| 91 | 203 | nordihydroguaiaretic acid | 13 | 1 µM | MCF7 | 0.618 | 0.177 | -0.163 |
| 92 | 1017 | fluphenazine | 506 | 10 µM | MCF7 | 0.618 | 0.155 | -0.185 |
| 93 | 204 | sulfasalazine | 13 | 100 µM | MCF7 | 0.616 | 0.195 | -0.144 |
| 94 | 169 | tacrolimus | 8 | 1 µM | MCF7 | 0.616 | 0.155 | -0.184 |
| 95 | 4 | metformin | 1 | 1 mM | MCF7 | 0.615 | 0.129 | -0.209 |
| 96 | 630 | colchicine | 109 | 1 µM | SKMEL5 | 0.613 | 0.206 | -0.131 |
| 97 | 842 | bucladesine | 504 | 2 µM | MCF7 | 0.607 | 0.176 | -0.158 |
| 98 | 762 | alpha-estradiol | 119 | 10 nM | MCF7 | 0.605 | 0.205 | -0.128 |
| 99 | 985 | fulvestrant | 506 | 1 µM | MCF7 | 0.602 | 0.165 | -0.166 |
| 100 | 611 | geldanamycin | 101 | 1 µM | MCF7 | 0.6 | 0.162 | -0.168 |
| 101 | 485 | deferoxamine | 67 | 100 µM | MCF7 | 0.595 | 0.204 | -0.123 |
| 102 | 594 | arachidonyltrifluoromethane | 95 | 10 µM | MCF7 | 0.587 | 0.174 | -0.149 |
| 103 | 345 | valproic acid | 33 | 10 mM | MCF7 | 0.584 | 0.137 | -0.184 |
| 104 | 667 | mercaptopurine | 116 | 10 µM | PC3 | 0.578 | 0.155 | -0.163 |
| 105 | 1112 | trichostatin A | 514 | 100 nM | MCF7 | 0.576 | 0.155 | -0.162 |
| 106 | 366 | imatinib | 36 | 10 µM | MCF7 | 0.573 | 0.164 | -0.151 |
| 107 | 606 | thalidomide | 98 | 100 µM | MCF7 | 0.571 | 0.17 | -0.144 |
| 108 | 1061 | nordihydroguaiaretic acid | 513 | 1 µM | MCF7 | 0.571 | 0.145 | -0.169 |
| 109 | 440 | W-13 | 58 | 10 µM | MCF7 | 0.562 | 0.176 | -0.133 |
| 110 | 369 | rosiglitazone | 37 | 10 µM | HL60 | 0.556 | 0.178 | -0.128 |
| 111 | 435 | novobiocin | 56 | 100 µM | PC3 | 0.555 | 0.161 | -0.144 |
| 112 | 202 | raloxifene | 13 | 100 nM | MCF7 | 0.555 | 0.152 | -0.153 |
| 113 | 258 | LY-294002 | 19 | 10 µM | MCF7 | 0.551 | 0.198 | -0.105 |
| 114 | 317 | N-phenylanthranilic acid | 25 | 10 µM | MCF7 | 0.547 | 0.191 | -0.11 |
| 115 | 264 | MK-886 | 20 | 1 µM | MCF7 | 0.547 | 0.157 | -0.144 |
| 116 | 998 | 17-allylamino-geldanamycin | 506 | 1 µM | MCF7 | 0.544 | 0.175 | -0.124 |
| 117 | 942 | prazosin | 502 | 10 µM | MCF7 | 0.542 | 0.139 | -0.159 |
| 118 | 143 | tamoxifen | 6 | 1 µM | MCF7 | 0.54 | 0.14 | -0.157 |
| 119 | 255 | dexamethasone | 19 | 1 µM | MCF7 | 0.536 | 0.171 | -0.124 |
| 120 | 142 | tolbutamide | 6 | 100 µM | MCF7 | 0.533 | 0.152 | -0.141 |
| 121 | 661 | splitomicin | 90 | 20 µM | PC3 | 0.531 | 0.145 | -0.147 |
| 122 | 665 | estradiol | 116 | 10 nM | PC3 | 0.531 | 0.139 | -0.153 |
| 123 | 991 | tretinoin | 506 | 1 µM | MCF7 | 0.525 | 0.174 | -0.115 |
| 124 | 609 | 5666823 | 101 | 100 µM | MCF7 | 0.513 | 0.117 | -0.165 |
| 125 | 702 | alpha-estradiol | 110b | 10 nM | PC3 | 0.511 | 0.154 | -0.127 |
| 126 | 575 | copper sulfate | 82 | 100 µM | MCF7 | 0.487 | 0.12 | -0.148 |
| 127 | 310 | fulvestrant | 23 | 10 nM | MCF7 | 0.46 | 0.119 | -0.134 |
| 128 | 590 | 3-aminobenzamide | 94 | 10 mM | MCF7 | 0.431 | 0.136 | -0.101 |
| 129 | 1043 | fulvestrant | 513 | 1 µM | MCF7 | 0 | 0.376 | 0.145 |
| 130 | 254 | SC-58125 | 18 | 10 µM | MCF7 | 0 | 0.354 | 0.154 |
| 131 | 966 | tretinoin | 502 | 1 µM | MCF7 | 0 | 0.339 | 0.155 |
| 132 | 622 | resveratrol | 107 | 50 µM | MCF7 | 0 | 0.327 | 0.188 |
| 133 | 403 | alpha-estradiol | 42 | 10 nM | ssMCF7 | 0 | 0.323 | 0.203 |
| 134 | 1004 | trifluoperazine | 506 | 10 µM | MCF7 | 0 | 0.32 | 0.295 |
| 135 | 906 | calmidazolium | 505 | 5 µM | MCF7 | 0 | 0.317 | 0.179 |
| 136 | 1054 | LY-294002 | 513 | 100 nM | MCF7 | 0 | 0.317 | 0.388 |
| 137 | 882 | ionomycin | 505 | 2 µM | MCF7 | 0 | 0.313 | 0.231 |
| 138 | 961 | 5253409 | 502 | 17 µM | MCF7 | 0 | 0.308 | 0.192 |
| 139 | 1041 | haloperidol | 513 | 10 µM | MCF7 | 0 | 0.306 | 0.274 |
| 140 | 1045 | sirolimus | 513 | 100 nM | MCF7 | 0 | 0.302 | 0.258 |
| 141 | 904 | 5109870 | 505 | 25 µM | MCF7 | 0 | 0.297 | 0.182 |
| 142 | 825 | rottlerin | 504 | 10 µM | MCF7 | 0 | 0.296 | 0.458 |
| 143 | 835 | carbamazepine | 504 | 100 nM | MCF7 | 0 | 0.296 | 0.399 |
| 144 | 965 | felodipine | 502 | 10 µM | MCF7 | 0 | 0.294 | 0.109 |
| 145 | 1049 | tretinoin | 513 | 1 µM | MCF7 | 0 | 0.293 | 0.153 |
| 146 | 833 | 5255229 | 504 | 13 µM | MCF7 | 0 | 0.293 | 0.185 |
| 147 | 460 | deferoxamine | 63 | 100 µM | PC3 | 0 | 0.288 | 0.156 |
| 148 | 413 | trichostatin A | 45 | 100 nM | ssMCF7 | 0 | 0.286 | 0.154 |
| 149 | 1048 | alpha-estradiol | 513 | 10 nM | MCF7 | 0 | 0.285 | 0.247 |
| 150 | 315 | acetylsalicylic acid | 25 | 100 µM | MCF7 | 0 | 0.279 | 0.169 |
| 151 | 993 | 17-dimethylamino-geldanamycin | 506 | 100 nM | MCF7 | 0 | 0.279 | 0.164 |
| 152 | 837 | blebbistatin | 504 | 17 µM | MCF7 | 0 | 0.278 | 0.224 |
| 153 | 971 | oxaprozin | 502 | 300 µM | MCF7 | 0 | 0.278 | 0.13 |
| 154 | 995 | prochlorperazine | 506 | 10 µM | MCF7 | 0 | 0.276 | 0.252 |
| 155 | 1015 | genistein | 506 | 10 µM | MCF7 | 0 | 0.276 | 0.228 |
| 156 | 363 | sodium phenylbutyrate | 35 | 100 µM | HL60 | 0 | 0.274 | 0.275 |
| 157 | 505 | 17-allylamino-geldanamycin | 73 | 1 µM | SKMEL5 | 0 | 0.273 | 0.185 |
| 158 | 574 | tetraethylenepentamine | 82 | 100 µM | MCF7 | 0 | 0.273 | 0.262 |
| 159 | 914 | rottlerin | 505 | 10 µM | MCF7 | 0 | 0.272 | 0.24 |
| 160 | 978 | pyrvinium | 502 | 1 µM | MCF7 | 0 | 0.272 | 0.157 |
| 161 | 863 | oxaprozin | 504 | 300 µM | MCF7 | 0 | 0.268 | 0.243 |
| 162 | 447 | tretinoin | 60 | 1 µM | PC3 | 0 | 0.268 | 0.276 |
| 163 | 166 | rofecoxib | 7 | 10 µM | MCF7 | 0 | 0.267 | 0.152 |
| 164 | 164 | dexverapamil | 7 | 10 µM | MCF7 | 0 | 0.264 | 0.235 |
| 165 | 506 | wortmannin | 73 | 10 nM | SKMEL5 | 0 | 0.263 | 0.428 |
| 166 | 402 | sirolimus | 42 | 100 nM | ssMCF7 | 0 | 0.261 | 0.253 |
| 167 | 986 | 17-allylamino-geldanamycin | 506 | 1 µM | MCF7 | 0 | 0.26 | 0.254 |
| 168 | 2 | metformin | 1 | 10 µM | MCF7 | 0 | 0.258 | 0.162 |
| 169 | 263 | clofibrate | 20 | 150 µM | MCF7 | 0 | 0.256 | 0.276 |
| 170 | 987 | sirolimus | 506 | 100 nM | MCF7 | 0 | 0.255 | 0.223 |
| 171 | 1012 | troglitazone | 506 | 10 µM | MCF7 | 0 | 0.255 | 0.188 |
| 172 | 404 | wortmannin | 42 | 10 nM | ssMCF7 | 0 | 0.253 | 0.212 |
| 173 | 443 | arachidonic acid | 59 | 10 µM | MCF7 | 0 | 0.252 | 0.256 |
| 174 | 944 | 5252917 | 502 | 14 µM | MCF7 | 0 | 0.251 | 0.328 |
| 175 | 624 | 4,5-dianilinophthalimide | 107 | 10 µM | MCF7 | 0 | 0.25 | 0.244 |
| 176 | 1059 | sirolimus | 513 | 100 nM | MCF7 | 0 | 0.248 | 0.15 |
| 177 | 1001 | sirolimus | 506 | 100 nM | MCF7 | 0 | 0.248 | 0.251 |
| 178 | 364 | trichostatin A | 35 | 100 nM | HL60 | 0 | 0.248 | 0.166 |
| 179 | 576 | novobiocin | 82 | 100 µM | MCF7 | 0 | 0.246 | 0.263 |
| 180 | 1080 | sirolimus | 513 | 100 nM | MCF7 | 0 | 0.246 | 0.229 |
| 181 | 381 | 17-allylamino-geldanamycin | 40 | 1 µM | MCF7 | 0 | 0.243 | 0.19 |
| 182 | 387 | estradiol | 41 | 10 nM | HL60 | 0 | 0.241 | 0.241 |
| 183 | 454 | cobalt chloride | 62 | 100 µM | MCF7 | 0 | 0.241 | 0.249 |
| 184 | 1013 | rosiglitazone | 506 | 10 µM | MCF7 | 0 | 0.241 | 0.23 |
| 185 | 952 | carbamazepine | 502 | 100 nM | MCF7 | 0 | 0.24 | 0.188 |
| 186 | 445 | diclofenac | 60 | 10 µM | PC3 | 0 | 0.24 | 0.29 |
| 187 | 959 | bucladesine | 502 | 2 µM | MCF7 | 0 | 0.24 | 0.286 |
| 188 | 579 | fisetin | 86 | 50 µM | PC3 | 0 | 0.239 | 0.123 |
| 189 | 459 | copper sulfate | 63 | 100 µM | PC3 | 0 | 0.238 | 0.146 |
| 190 | 521 | 17-allylamino-geldanamycin | 74 | 1 µM | ssMCF7 | 0 | 0.237 | 0.147 |
| 191 | 896 | 5152487 | 505 | 10 µM | MCF7 | 0 | 0.236 | 0.186 |
| 192 | 841 | resveratrol | 504 | 10 µM | MCF7 | 0 | 0.234 | 0.188 |
| 193 | 161 | verapamil | 7a | 10 µM | MCF7 | 0 | 0.234 | 0.209 |
| 194 | 1007 | LY-294002 | 506 | 10 µM | MCF7 | 0 | 0.232 | 0.182 |
| 195 | 1044 | 17-allylamino-geldanamycin | 513 | 1 µM | MCF7 | 0 | 0.23 | 0.147 |
| 196 | 828 | 5252917 | 504 | 14 µM | MCF7 | 0 | 0.223 | 0.207 |
| 197 | 954 | blebbistatin | 502 | 17 µM | MCF7 | 0 | 0.223 | 0.192 |
| 198 | 836 | monorden | 504 | 100 nM | MCF7 | 0 | 0.222 | 0.152 |
| 199 | 439 | oxamic acid | 58 | 10 mM | MCF7 | 0 | 0.221 | 0.212 |
| 200 | 1114 | tyrphostin AG-825 | 514 | 25 µM | MCF7 | 0 | 0.221 | 0.236 |
| 201 | 312 | staurosporine | 24 | 1 µM | MCF7 | 0 | 0.218 | 0.136 |
| 202 | 371 | rofecoxib | 37 | 10 µM | HL60 | 0 | 0.218 | 0.124 |
| 203 | 487 | pirinixic acid | 67 | 100 µM | MCF7 | 0 | 0.218 | 0.2 |
| 204 | 1051 | 17-dimethylamino-geldanamycin | 513 | 100 nM | MCF7 | 0 | 0.217 | 0.32 |
| 205 | 419 | chlorpromazine | 46 | 10 µM | MCF7 | 0 | 0.214 | 0.219 |
| 206 | 334 | mercaptopurine | 28 | 100 µM | MCF7 | 0 | 0.212 | 0.296 |
| 207 | 418 | haloperidol | 46 | 10 µM | MCF7 | 0 | 0.211 | 0.215 |
| 208 | 832 | Y-27632 | 504 | 3 µM | MCF7 | 0 | 0.21 | 0.148 |
| 209 | 582 | butein | 87 | 10 µM | PC3 | 0 | 0.209 | 0.315 |
| 210 | 362 | sirolimus | 35 | 100 nM | HL60 | 0 | 0.209 | 0.349 |
| 211 | 508 | staurosporine | 73 | 10 nM | SKMEL5 | 0 | 0.207 | 0.272 |
| 212 | 224 | tretinoin | 17 | 1 µM | MCF7 | 0 | 0.205 | 0.156 |
| 213 | 1064 | 17-allylamino-geldanamycin | 513 | 1 µM | MCF7 | 0 | 0.203 | 0.204 |
| 214 | 874 | depudecin | 504 | 1 µM | MCF7 | 0 | 0.202 | 0.192 |
| 215 | 1115 | phenanthridinone | 514 | 51 µM | MCF7 | 0 | 0.202 | 0.225 |
| 216 | 453 | indometacin | 62 | 100 µM | MCF7 | 0 | 0.202 | 0.261 |
| 217 | 910 | trifluoperazine | 505 | 10 µM | MCF7 | 0 | 0.201 | 0.152 |
| 218 | 425 | staurosporine | 54 | 10 nM | MCF7 | 0 | 0.2 | 0.149 |
| 219 | 976 | 5182598 | 502 | 25 µM | MCF7 | 0 | 0.199 | 0.187 |
| 220 | 1005 | 17-allylamino-geldanamycin | 506 | 1 µM | MCF7 | 0 | 0.199 | 0.235 |
| 221 | 448 | trichostatin A | 60 | 100 nM | PC3 | 0 | 0.198 | 0.22 |
| 222 | 367 | fulvestrant | 36 | 1 µM | MCF7 | 0 | 0.198 | 0.113 |
| 223 | 1075 | fluphenazine | 513 | 10 µM | MCF7 | 0 | 0.197 | 0.207 |
| 224 | 498 | tetraethylenepentamine | 70 | 100 µM | ssMCF7 | 0 | 0.196 | 0.157 |
| 225 | 908 | 5140203 | 505 | 15 µM | MCF7 | 0 | 0.195 | 0.142 |
| 226 | 703 | genistein | 110b | 10 µM | PC3 | 0 | 0.194 | 0.199 |
| 227 | 573 | deferoxamine | 82 | 100 µM | MCF7 | 0 | 0.194 | 0.253 |
| 228 | 382 | genistein | 40 | 10 µM | MCF7 | 0 | 0.194 | 0.248 |
| 229 | 887 | celastrol | 505 | 3 µM | MCF7 | 0 | 0.194 | 0.113 |
| 230 | 417 | thioridazine | 46 | 10 µM | MCF7 | 0 | 0.193 | 0.193 |
| 231 | 640 | paclitaxel | 111 | 100 nM | MCF7 | 0 | 0.193 | 0.21 |
| 232 | 1042 | acetylsalicylic acid | 513 | 100 µM | MCF7 | 0 | 0.192 | 0.176 |
| 233 | 1047 | valproic acid | 513 | 1 mM | MCF7 | 0 | 0.19 | 0.126 |
| 234 | 608 | NU-1025 | 98 | 100 µM | MCF7 | 0 | 0.189 | 0.208 |
| 235 | 901 | 5114445 | 505 | 10 µM | MCF7 | 0 | 0.187 | 0.131 |
| 236 | 331 | trichostatin A | 28 | 100 nM | MCF7 | 0 | 0.186 | 0.188 |
| 237 | 629 | valproic acid | 109 | 1 mM | SKMEL5 | 0 | 0.182 | 0.125 |
| 238 | 1074 | LY-294002 | 513 | 10 µM | MCF7 | 0 | 0.181 | 0.229 |
| 239 | 831 | 17-allylamino-geldanamycin | 504 | 1 µM | MCF7 | 0 | 0.179 | 0.187 |
| 240 | 141 | chlorpropamide | 6 | 100 µM | MCF7 | 0 | 0.179 | 0.119 |
| 241 | 389 | wortmannin | 41 | 10 nM | HL60 | 0 | 0.178 | 0.206 |
| 242 | 917 | quercetin | 505 | 1 µM | MCF7 | 0 | 0.177 | 0.15 |
| 243 | 921 | sirolimus | 505 | 100 nM | MCF7 | 0 | 0.176 | 0.199 |
| 244 | 612 | LM-1685 | 101 | 10 µM | MCF7 | 0 | 0.175 | 0.324 |
| 245 | 643 | W-13 | 112 | 10 µM | MCF7 | 0 | 0.174 | 0.142 |
| 246 | 583 | HNMPA-(AM)3 | 87 | 5 µM | PC3 | 0 | 0.173 | 0.255 |
| 247 | 380 | tamoxifen | 39 | 1 µM | MCF7 | 0 | 0.173 | 0.144 |
| 248 | 458 | valproic acid | 63 | 1 mM | PC3 | 0 | 0.172 | 0.164 |
| 249 | 412 | tetraethylenepentamine | 44 | 100 µM | HL60 | 0 | 0.171 | 0.207 |
| 250 | 61 | metformin | 2a | 10 µM | MCF7 | 0 | 0.171 | 0.127 |
| 251 | 124 | mesalazine | 5 | 100 µM | MCF7 | 0 | 0.168 | 0.312 |
| 252 | 922 | celecoxib | 505 | 10 µM | MCF7 | 0 | 0.168 | 0.203 |
| 253 | 960 | 5279552 | 502 | 22 µM | MCF7 | 0 | 0.167 | 0.193 |
| 254 | 486 | calmidazolium | 67 | 5 µM | MCF7 | 0 | 0.166 | 0.177 |
| 255 | 336 | nitrendipine | 29 | 10 µM | MCF7 | 0 | 0.165 | 0.106 |
| 256 | 602 | ciclosporin | 96 | 1 µM | MCF7 | 0 | 0.164 | 0.207 |
| 257 | 593 | geldanamycin | 95 | 1 µM | MCF7 | 0 | 0.164 | 0.145 |
| 258 | 3 | metformin | 1 | 100 nM | MCF7 | 0 | 0.162 | 0.258 |
| 259 | 989 | valproic acid | 506 | 1 mM | MCF7 | 0 | 0.162 | 0.157 |
| 260 | 972 | geldanamycin | 502 | 1 µM | MCF7 | 0 | 0.161 | 0.208 |
| 261 | 267 | genistein | 21 | 1 µM | MCF7 | 0 | 0.159 | 0.196 |
| 262 | 870 | pyrvinium | 504 | 1 µM | MCF7 | 0 | 0.158 | 0.196 |
| 263 | 446 | 15-delta prostaglandin J2 | 60 | 10 µM | PC3 | 0 | 0.157 | 0.261 |
| 264 | 122 | alpha-estradiol | 5 | 10 nM | MCF7 | 0 | 0.157 | 0.196 |
| 265 | 668 | monastrol | 116 | 100 µM | PC3 | 0 | 0.156 | 0.21 |
| 266 | 979 | ionomycin | 502 | 2 µM | MCF7 | 0 | 0.153 | 0.268 |
| 267 | 604 | arachidonic acid | 96 | 10 µM | MCF7 | 0 | 0.15 | 0.304 |
| 268 | 370 | troglitazone | 37 | 10 µM | HL60 | 0 | 0.148 | 0.083 |
| 269 | 365 | estradiol | 36 | 100 nM | MCF7 | 0 | 0.148 | 0.128 |
| 270 | 437 | novobiocin | 58 | 100 µM | MCF7 | 0 | 0.146 | 0.115 |
| 271 | 642 | tioguanine | 112 | 10 µM | MCF7 | 0 | 0.146 | 0.202 |
| 272 | 390 | tretinoin | 41 | 1 µM | HL60 | 0 | 0.146 | 0.157 |
| 273 | 361 | LY-294002 | 35 | 10 µM | HL60 | 0 | 0.146 | 0.151 |
| 274 | 388 | raloxifene | 41 | 100 nM | HL60 | 0 | 0.145 | 0.397 |
| 275 | 1081 | wortmannin | 513 | 10 nM | MCF7 | 0 | 0.145 | 0.191 |
| 276 | 507 | SC-58125 | 73 | 10 µM | SKMEL5 | 0 | 0.145 | 0.237 |
| 277 | 405 | tetraethylenepentamine | 43 | 10 µM | MCF7 | 0 | 0.144 | 0.174 |
| 278 | 383 | cobalt chloride | 40 | 100 µM | MCF7 | 0 | 0.143 | 0.235 |
| 279 | 584 | dimethyloxalylglycine | 87 | 1 mM | PC3 | 0 | 0.141 | 0.171 |
| 280 | 432 | 17-allylamino-geldanamycin | 55 | 1 µM | PC3 | 0 | 0.139 | 0.139 |
| 281 | 484 | monorden | 66 | 100 nM | PC3 | 0 | 0.135 | 0.231 |
| 282 | 504 | troglitazone | 71 | 10 µM | SKMEL5 | 0 | 0.134 | 0.284 |
| 283 | 208 | SC-58125 | 16 | 10 µM | MCF7 | 0 | 0.13 | 0.105 |
| 284 | 429 | LY-294002 | 55 | 10 µM | PC3 | 0 | 0.127 | 0.23 |
| 285 | 494 | fluphenazine | 69 | 10 µM | SKMEL5 | 0 | 0.125 | 0.156 |
| 286 | 541 | gefitinib | 75 | 10 µM | HL60 | 0 | 0.124 | 0.302 |
| 287 | 436 | fasudil | 56 | 10 µM | PC3 | 0 | 0.123 | 0.14 |
| 288 | 450 | 17-allylamino-geldanamycin | 61 | 1 µM | PC3 | 0 | 0.121 | 0.229 |
| 289 | 409 | valproic acid | 44 | 1 mM | HL60 | 0 | 0.112 | 0.211 |
| 290 | 496 | iloprost | 69 | 1 µM | SKMEL5 | 0 | 0.11 | 0.095 |
| 291 | 662 | resveratrol | 90 | 50 µM | PC3 | 0 | 0.103 | 0.245 |
| 292 | 783 | colforsin | 120 | 50 µM | HL60 | 0 | 0.084 | 0.208 |
| 293 | 666 | butirosin | 116 | 10 µM | PC3 | 0 | -0.115 | -0.176 |
| 294 | 457 | tetraethylenepentamine | 63 | 100 µM | PC3 | 0 | -0.128 | -0.148 |
| 295 | 168 | sulindac | 8 | 100 µM | MCF7 | 0 | -0.129 | -0.153 |
| 296 | 313 | NU-1025 | 24 | 100 µM | MCF7 | 0 | -0.13 | -0.296 |
| 297 | 892 | 5162773 | 505 | 7 µM | MCF7 | 0 | -0.133 | -0.291 |
| 298 | 462 | troglitazone | 65 | 10 µM | PC3 | 0 | -0.135 | -0.218 |
| 299 | 499 | novobiocin | 70 | 100 µM | ssMCF7 | 0 | -0.135 | -0.272 |
| 300 | 421 | trifluoperazine | 53 | 10 µM | MCF7 | 0 | -0.139 | -0.248 |
| 301 | 444 | clofibrate | 59 | 100 µM | MCF7 | 0 | -0.139 | -0.199 |
| 302 | 903 | 5151277 | 505 | 14 µM | MCF7 | 0 | -0.139 | -0.171 |
| 303 | 121 | estradiol | 5 | 10 nM | MCF7 | 0 | -0.142 | -0.165 |
| 304 | 281 | fludrocortisone | 22a | 1 µM | MCF7 | 0 | -0.143 | -0.177 |
| 305 | 1140 | MG-132 | 514 | 21 µM | MCF7 | 0 | -0.144 | -0.234 |
| 306 | 144 | chlorpropamide | 6 | 100 µM | MCF7 | 0 | -0.145 | -0.102 |
| 307 | 314 | exisulind | 24 | 50 µM | MCF7 | 0 | -0.147 | -0.319 |
| 308 | 489 | monorden | 68 | 100 nM | MCF7 | 0 | -0.147 | -0.121 |
| 309 | 844 | 5253409 | 504 | 17 µM | MCF7 | 0 | -0.148 | -0.132 |
| 310 | 308 | sulindac sulfide | 23 | 50 µM | MCF7 | 0 | -0.15 | -0.138 |
| 311 | 346 | valproic acid | 33 | 2 mM | MCF7 | 0 | -0.151 | -0.134 |
| 312 | 311 | monastrol | 24 | 100 µM | MCF7 | 0 | -0.151 | -0.331 |
| 313 | 379 | cobalt chloride | 39 | 100 µM | MCF7 | 0 | -0.151 | -0.125 |
| 314 | 283 | quercetin | 22a | 1 µM | MCF7 | 0 | -0.153 | -0.202 |
| 315 | 482 | celecoxib | 66 | 10 µM | PC3 | 0 | -0.154 | -0.189 |
| 316 | 266 | thalidomide | 21 | 100 µM | MCF7 | 0 | -0.157 | -0.133 |
| 317 | 544 | monorden | 75 | 100 nM | HL60 | 0 | -0.158 | -0.196 |
| 318 | 284 | tacrolimus | 22a | 1 µM | MCF7 | 0 | -0.161 | -0.263 |
| 319 | 375 | tamoxifen | 38 | 1 µM | ssMCF7 | 0 | -0.162 | -0.147 |
| 320 | 328 | LY-294002 | 26b | 10 µM | MCF7 | 0 | -0.163 | -0.118 |
| 321 | 644 | colchicine | 112 | 100 nM | MCF7 | 0 | -0.166 | -0.102 |
| 322 | 868 | 5182598 | 504 | 25 µM | MCF7 | 0 | -0.166 | -0.231 |
| 323 | 848 | felodipine | 504 | 10 µM | MCF7 | 0 | -0.167 | -0.219 |
| 324 | 1021 | estradiol | 506 | 10 nM | MCF7 | 0 | -0.17 | -0.269 |
| 325 | 337 | felodipine | 29 | 10µM | MCF7 | 0 | -0.174 | -0.175 |
| 326 | 862 | 5230742 | 504 | 17 ¬µM | MCF7 | 0 | -0.174 | -0.216 |
| 327 | 994 | valproic acid | 506 | 200 µM | MCF7 | 0 | -0.175 | -0.148 |
| 328 | 627 | monastrol | 108 | 100 µM | MCF7 | 0 | -0.176 | -0.276 |
| 329 | 543 | 1,5-isoquinolinediol | 75 | 100 µM | HL60 | 0 | -0.176 | -0.21 |
| 330 | 422 | thioridazine | 53 | 1 µM | MCF7 | 0 | -0.177 | -0.208 |
| 331 | 1105 | monensin | 514 | 11 µM | MCF7 | 0 | -0.177 | -0.183 |
| 332 | 451 | TTNPB | 61 | 100 nM | PC3 | 0 | -0.178 | -0.136 |
| 333 | 1 | metformin | 1 | 10 µM | MCF7 | 0 | -0.179 | -0.212 |
| 334 | 424 | LY-294002 | 53 | 100 nM | MCF7 | 0 | -0.181 | -0.342 |
| 335 | 21 | phenformin | 2 | 10 µM | MCF7 | 0 | -0.184 | -0.151 |
| 336 | 1063 | 17-allylamino-geldanamycin | 513 | 1 µM | MCF7 | 0 | -0.185 | -0.204 |
| 337 | 500 | copper sulfate | 70 | 100 µM | ssMCF7 | 0 | -0.185 | -0.27 |
| 338 | 438 | copper sulfate | 58 | 100 µM | MCF7 | 0 | -0.187 | -0.179 |
| 339 | 332 | trichostatin A | 28 | 100 nM | MCF7 | 0 | -0.187 | -0.111 |
| 340 | 1082 | haloperidol | 513 | 10 µM | MCF7 | 0 | -0.187 | -0.192 |
| 341 | 347 | valproic acid | 33 | 500 µM | MCF7 | 0 | -0.19 | -0.179 |
| 342 | 596 | monastrol | 95 | 100 µM | MCF7 | 0 | -0.191 | -0.191 |
| 343 | 407 | sodium phenylbutyrate | 43 | 1 mM | MCF7 | 0 | -0.197 | -0.174 |
| 344 | 1079 | estradiol | 513 | 10 nM | MCF7 | 0 | -0.198 | -0.18 |
| 345 | 869 | wortmannin | 504 | 1 µM | MCF7 | 0 | -0.198 | -0.185 |
| 346 | 843 | 5279552 | 504 | 22 µM | MCF7 | 0 | -0.202 | -0.26 |
| 347 | 348 | valproic acid | 33 | 50 µM | MCF7 | 0 | -0.206 | -0.224 |
| 348 | 591 | bucladesine | 94 | 20 µM | MCF7 | 0 | -0.206 | -0.163 |
| 349 | 638 | genistein | 111 | 10 µM | MCF7 | 0 | -0.21 | -0.157 |
| 350 | 1019 | LY-294002 | 506 | 10 µM | MCF7 | 0 | -0.212 | -0.14 |
| 351 | 1020 | valproic acid | 506 | 500 µM | MCF7 | 0 | -0.212 | -0.247 |
| 352 | 839 | 5224221 | 504 | 12 µM | MCF7 | 0 | -0.214 | -0.186 |
| 353 | 542 | SC-58125 | 75 | 10 µM | HL60 | 0 | -0.215 | -0.214 |
| 354 | 911 | wortmannin | 505 | 10 nM | MCF7 | 0 | -0.215 | -0.182 |
| 355 | 343 | fasudil | 31 | 10 µM | MCF7 | 0 | -0.218 | -0.228 |
| 356 | 1113 | doxycycline | 514 | 14 µM | MCF7 | 0 | -0.219 | -0.203 |
| 357 | 641 | benserazide | 112 | 10 µM | MCF7 | 0 | -0.22 | -0.228 |
| 358 | 341 | sodium phenylbutyrate | 31 | 100 µM | MCF7 | 0 | -0.22 | -0.126 |
| 359 | 327 | arachidonyltrifluoromethane | 26b | 10 µM | MCF7 | 0 | -0.222 | -0.272 |
| 360 | 996 | LY-294002 | 506 | 100 nM | MCF7 | 0 | -0.227 | -0.175 |
| 361 | 982 | depudecin | 502 | 1 µM | MCF7 | 0 | -0.229 | -0.198 |
| 362 | 915 | topiramate | 505 | 3 µM | MCF7 | 0 | -0.229 | -0.183 |
| 363 | 483 | imatinib | 66 | 10 µM | PC3 | 0 | -0.232 | -0.141 |
| 364 | 1135 | minocycline | 514 | 11 µM | MCF7 | 0 | -0.233 | -0.148 |
| 365 | 592 | probucol | 94 | 10 µM | MCF7 | 0 | -0.234 | -0.138 |
| 366 | 974 | ikarugamycin | 502 | 2 µM | MCF7 | 0 | -0.243 | -0.148 |
| 367 | 406 | tetraethylenepentamine | 43 | 100 µM | MCF7 | 0 | -0.245 | -0.118 |
| 368 | 834 | 5211181 | 504 | 12 µM | MCF7 | 0 | -0.247 | -0.194 |
| 369 | 325 | monorden | 26b | 100 nM | MCF7 | 0 | -0.25 | -0.252 |
| 370 | 1138 | phentolamine | 514 | 12 µM | MCF7 | 0 | -0.253 | -0.172 |
| 371 | 890 | 5149715 | 505 | 10 µM | MCF7 | 0 | -0.253 | -0.2 |
| 372 | 307 | sulindac | 23 | 50 µM | MCF7 | 0 | -0.257 | -0.106 |
| 373 | 374 | dexamethasone | 38 | 1 µM | ssMCF7 | 0 | -0.259 | -0.193 |
| 374 | 410 | valproic acid | 44 | 10 mM | HL60 | 0 | -0.26 | -0.239 |
| 375 | 1071 | rosiglitazone | 513 | 10 µM | MCF7 | 0 | -0.268 | -0.291 |
| 376 | 22 | phenyl biguanide | 2 | 10 µM | MCF7 | 0 | -0.284 | -0.132 |
| 377 | 488 | iloprost | 67 | 1 µM | MCF7 | 0 | -0.297 | -0.227 |
| 378 | 207 | LM-1685 | 16 | 10 µM | MCF7 | -0.382 | -0.132 | 0.081 |
| 379 | 663 | U0125 | 90 | 1 µM | PC3 | -0.425 | -0.134 | 0.103 |
| 380 | 497 | valproic acid | 70 | 1 mM | ssMCF7 | -0.434 | -0.146 | 0.096 |
| 381 | 603 | nifedipine | 96 | 10 µM | MCF7 | -0.465 | -0.118 | 0.141 |
| 382 | 123 | dexamethasone | 5 | 1 µM | MCF7 | -0.488 | -0.111 | 0.161 |
| 383 | 564 | 15-delta prostaglandin J2 | 79 | 10 µM | SKMEL5 | -0.49 | -0.129 | 0.144 |
| 384 | 481 | pirinixic acid | 66 | 100 µM | PC3 | -0.492 | -0.128 | 0.146 |
| 385 | 463 | rofecoxib | 65 | 10 µM | PC3 | -0.494 | -0.122 | 0.153 |
| 386 | 782 | estradiol | 120 | 10 nM | HL60 | -0.503 | -0.117 | 0.163 |
| 387 | 461 | LY-294002 | 65 | 10 µM | PC3 | -0.51 | -0.096 | 0.188 |
| 388 | 265 | prednisolone | 21 | 1 µM | MCF7 | -0.526 | -0.182 | 0.111 |
| 389 | 309 | exisulind | 23 | 50 µM | MCF7 | -0.528 | -0.164 | 0.13 |
| 390 | 23 | valproic acid | 2 | 1 mM | MCF7 | -0.53 | -0.15 | 0.145 |
| 391 | 1108 | 12,13-EODE | 514 | 200 nM | MCF7 | -0.533 | -0.169 | 0.128 |
| 392 | 503 | indometacin | 71 | 100 µM | SKMEL5 | -0.535 | -0.202 | 0.096 |
| 393 | 464 | pirinixic acid | 65 | 100 µM | PC3 | -0.553 | -0.161 | 0.147 |
| 394 | 607 | butein | 98 | 10 µM | MCF7 | -0.557 | -0.144 | 0.166 |
| 395 | 326 | sirolimus | 26b | 100 nM | MCF7 | -0.557 | -0.192 | 0.118 |
| 396 | 916 | 17-allylamino-geldanamycin | 505 | 1 µM | MCF7 | -0.562 | -0.162 | 0.151 |
| 397 | 502 | sodium phenylbutyrate | 71 | 200 µM | SKMEL5 | -0.564 | -0.095 | 0.219 |
| 398 | 1066 | geldanamycin | 513 | 1 µM | MCF7 | -0.569 | -0.183 | 0.134 |
| 399 | 1002 | valproic acid | 506 | 50 µM | MCF7 | -0.575 | -0.162 | 0.158 |
| 400 | 427 | iloprost | 54 | 1 µM | MCF7 | -0.576 | -0.194 | 0.127 |
| 401 | 605 | monastrol | 98 | 20 µM | MCF7 | -0.582 | -0.178 | 0.146 |
| 402 | 430 | rosiglitazone | 55 | 10 µM | PC3 | -0.583 | -0.221 | 0.104 |
| 403 | 431 | troglitazone | 55 | 10 µM | PC3 | -0.585 | -0.142 | 0.184 |
| 404 | 256 | rofecoxib | 19 | 10 µM | MCF7 | -0.585 | -0.197 | 0.129 |
| 405 | 919 | carbamazepine | 505 | 100 nM | MCF7 | -0.587 | -0.164 | 0.163 |
| 406 | 881 | docosahexaenoic acid ethyl ester | 505 | 100 µM | MCF7 | -0.603 | -0.174 | 0.162 |
| 407 | 885 | 5186223 | 505 | 12 µM | MCF7 | -0.605 | -0.231 | 0.106 |
| 408 | 316 | flufenamic acid | 25 | 10 µM | MCF7 | -0.609 | -0.161 | 0.178 |
| 409 | 631 | benserazide | 109 | 10 µM | SKMEL5 | -0.616 | -0.141 | 0.202 |
| 410 | 1008 | geldanamycin | 506 | 1 µM | MCF7 | -0.618 | -0.169 | 0.175 |
| 411 | 342 | novobiocin | 31 | 100 µM | MCF7 | -0.636 | -0.182 | 0.172 |
| 412 | 905 | clotrimazole | 505 | 50 µM | MCF7 | -0.637 | -0.219 | 0.136 |
| 413 | 900 | 5186324 | 505 | 2 µM | MCF7 | -0.641 | -0.236 | 0.121 |
| 414 | 920 | decitabine | 505 | 100 nM | MCF7 | -0.643 | -0.251 | 0.107 |
| 415 | 493 | monorden | 69 | 100 nM | SKMEL5 | -0.645 | -0.147 | 0.212 |
| 416 | 970 | 5230742 | 502 | 17 µM | MCF7 | -0.645 | -0.164 | 0.195 |
| 417 | 205 | rofecoxib | 16 | 10 µM | MCF7 | -0.646 | -0.14 | 0.22 |
| 418 | 1024 | haloperidol | 506 | 10 µM | MCF7 | -0.654 | -0.135 | 0.229 |
| 419 | 849 | tretinoin | 504 | 1 µM | MCF7 | -0.655 | -0.234 | 0.131 |
| 420 | 434 | sodium phenylbutyrate | 56 | 1 mM | PC3 | -0.661 | -0.16 | 0.208 |
| 421 | 1119 | yohimbine | 514 | 23 µM | MCF7 | -0.664 | -0.158 | 0.212 |
| 422 | 1109 | 3-hydroxy-DL-kynurenine | 514 | 9 µM | MCF7 | -0.675 | -0.163 | 0.213 |
| 423 | 411 | sodium phenylbutyrate | 44 | 1 mM | HL60 | -0.688 | -0.137 | 0.246 |
| 424 | 664 | docosahexaenoic acid ethyl ester | 90 | 100 µM | PC3 | -0.688 | -0.151 | 0.232 |
| 425 | 441 | arachidonic acid | 59 | 10 µM | MCF7 | -0.688 | -0.211 | 0.172 |
| 426 | 201 | 15-delta prostaglandin J2 | 13 | 10 µM | MCF7 | -0.689 | -0.193 | 0.191 |
| 427 | 373 | estradiol | 38 | 10 nM | ssMCF7 | -0.689 | -0.199 | 0.185 |
| 428 | 449 | monorden | 61 | 100 nM | PC3 | -0.7 | -0.189 | 0.201 |
| 429 | 495 | pirinixic acid | 69 | 100 µM | SKMEL5 | -0.702 | -0.165 | 0.226 |
| 430 | 376 | raloxifene | 38 | 100 nM | ssMCF7 | -0.709 | -0.273 | 0.122 |
| 431 | 501 | LY-294002 | 71 | 10 µM | SKMEL5 | -0.713 | -0.18 | 0.217 |
| 432 | 681 | monastrol | 117 | 100 µM | MCF7 | -0.72 | -0.175 | 0.226 |
| 433 | 1078 | valproic acid | 513 | 500 µM | MCF7 | -0.72 | -0.199 | 0.202 |
| 434 | 871 | ionomycin | 504 | 2 µM | MCF7 | -0.74 | -0.134 | 0.278 |
| 435 | 167 | amitriptyline | 8 | 1 µM | MCF7 | -0.74 | -0.172 | 0.24 |
| 436 | 206 | celecoxib | 16 | 10 µM | MCF7 | -0.745 | -0.19 | 0.225 |
| 437 | 491 | dopamine | 68 | 1 µM | MCF7 | -0.747 | -0.14 | 0.276 |
| 438 | 492 | haloperidol | 68 | 10 µM | MCF7 | -0.747 | -0.219 | 0.197 |
| 439 | 344 | 2-deoxy-D-glucose | 31 | 10 mM | MCF7 | -0.749 | -0.243 | 0.174 |
| 440 | 918 | ikarugamycin | 505 | 2 µM | MCF7 | -0.75 | -0.167 | 0.251 |
| 441 | 1101 | (-)-catechin | 514 | 11 µM | MCF7 | -0.754 | -0.151 | 0.269 |
| 442 | 442 | oligomycin | 59 | 1 µM | MCF7 | -0.756 | -0.15 | 0.271 |
| 443 | 268 | genistein | 21 | 1 µM | MCF7 | -0.768 | -0.206 | 0.222 |
| 444 | 614 | monastrol | 103 | 20 µM | MCF7 | -0.77 | -0.217 | 0.212 |
| 445 | 1132 | BW-B70C | 514 | 32 µM | MCF7 | -0.822 | -0.205 | 0.253 |
| 446 | 333 | diclofenac | 28 | 10 µM | MCF7 | -0.822 | -0.223 | 0.235 |
| 447 | 408 | sodium phenylbutyrate | 43 | 1 mM | MCF7 | -0.824 | -0.257 | 0.202 |
| 448 | 490 | fluphenazine | 68 | 10 µM | MCF7 | -0.829 | -0.158 | 0.304 |
| 449 | 1121 | DL-PPMP | 514 | 2 µM | MCF7 | -0.833 | -0.261 | 0.203 |
| 450 | 601 | MK-886 | 96 | 1 µM | MCF7 | -0.855 | -0.158 | 0.318 |
| 451 | 452 | indometacin | 61 | 100 µM | PC3 | -0.856 | -0.244 | 0.233 |
| 452 | 1141 | tyrphostin AG-1478 | 514 | 32 µM | MCF7 | -0.921 | -0.229 | 0.284 |
| 453 | 864 | geldanamycin | 504 | 1 µM | MCF7 | -1 | -0.244 | 0.313 |
